# Mechanism of Guigan Longmu Decoction in the Treatment of Arrhythmias **Based on Network Pharmacology and Untargeted Metabolomics Assays**

**DOI:** 10.2174/0113862073293313240519161145

**Published:** 2024-06-03

**Authors:** Tuo Deng, Sheng Guo, Jie Yang, Xiao-yu Huang, Xiao-bin Lu, Jing Lu, Liang Liu, Ze Du

**Affiliations:** 1 Hubei Provincial Hospital of Traditional Chinese Medicine, Wuhan, Hubei, 430061, China;; 2 Hubei University of Chinese Medicine, Wuhan, Hubei, 430065, China

**Keywords:** Arrhythmias, guigan longmu decoction, network pharmacology, widely-targeted metabolism quantification analysis, untargeted metabolomics, molecular docking

## Abstract

**Background:**

Guigan longmu decoction (GGLM), a traditional Chinese medicine compound, has demonstrated efficacy in treating rapid arrhythmia clinically. Nevertheless, its mechanism of action remains elusive. This study aims to elucidate the molecular mechanism underlying the efficacy of GGLM in treating arrhythmia utilizing non-targeted metabolomics, widely-targeted metabolomics, and network pharmacology, subsequently validated through animal experiments.

**Methods:**

Initially, network pharmacology analysis and widely-targeted metabolomics were performed on GGLM. Subsequent to that, rats were administered GGLM intervention, and non-targeted metabolomics assays were utilized to identify metabolites in rat plasma post-administration. The primary signaling pathways, core targets, and key active ingredients of GGLM influencing arrhythmia were identified. Additionally, to validate the therapeutic efficacy of GGLM on arrhythmia rat models, a rat model of rapid arrhythmia was induced *via* subcutaneous injection of isoproterenol, and alterations in pertinent pathogenic pathways and proteins in the rat model were assessed through qRT-PCR and Western blot following GGLM administration.

**Results:**

The results of network pharmacology showed that 99 active ingredients in GGLM acted on 249 targets and 201 signaling pathways, which may be key to treating arrhythmia. Widely-targeted metabolic quantification analysis detected a total of 448 active ingredients in GGLM, while non-targeted metabolomics identified 279 different metabolites and 10 major metabolic pathways in rats. A comprehensive analysis of the above results revealed that the core key active ingredients of GGLM in treating arrhythmia include calycosin, licochalcone B, glabridin, naringenin, medicarpin, formononetin, quercetin, isoliquiritigenin, and resveratrol. These active ingredients mainly act on the relevant molecules and proteins upstream and downstream of the MAPK pathway to delay the onset of arrhythmia. Animal experimental results showed that the heart rate of rats in the model group increased significantly, and the mRNA and protein expression of p38, MAPK, JNK, ERK, NF-kb, IL-1β, and IL-12 in myocardial tissue also increased significantly. However, after intervention with GGLM, the heart rate of rats in the drug group decreased significantly, while the mRNA and protein expression of p38 MAPK, JNK, ERK1, NF-kb, IL-1β, and IL-12 in myocardial tissue decreased significantly.

**Conclusion:**

GGLM, as an adjunctive therapy in traditional Chinese medicine, exhibits favorable therapeutic efficacy against arrhythmia. This can be attributed to the abundant presence of bioactive compounds in the formulation, including verminin, glycyrrhizin B, glabridine, naringenin, ononin, quercetin, isorhamnetin, and kaempferol. The metabolites derived from these active ingredients have the potential to mitigate myocardial inflammation and decelerate heart rate by modulating the expression of proteins associated with the MAPK signaling pathway *in vivo*.

## 1 INTRODUCTION

Arrhythmia is a common cardiovascular disease with a high incidence in clinical practice. It can occur alone or in conjunction with other diseases and has become a major issue in the healthcare field. Arrhythmia is a significant cause of global morbidity and mortality, accounting for 10% to 15% of all deaths [1]. A considerable portion of the approximately 3.7 million cases of Sudden Cardiac Death (SCD) worldwide each year are attributed to arrhythmias [2]. Recent studies have shown that hereditary arrhythmias are one of the main causes of SCD in young people. The reported incidence of arrhythmia-induced SCD in North America and Europe is 50 to 100 per 100000 [3-5]. Estimating the incidence of arrhythmia is more complex due to the fact that a significant portion of ventricular fibrillation and ventricular tachycardia are caused by structural heart disease, such as coronary artery atherosclerosis [6]. The occurrence of arrhythmias is complex, involving anatomical structures, ion channels, regulatory proteins, and interactions among cells, cardiomyocytes, fibroblasts, and the immune system [7]. However, a thorough understanding of the mechanisms of arrhythmia and how antiarrhythmic drugs affect these mechanisms is only the first step in appropriately managing arrhythmias. In drug selection and monitoring, clinical factors, side effects, and the risk of proarrhythmia are often more important than the mechanisms of arrhythmia [8].

Currently, pharmaceutical treatments for arrhythmia in clinical practice have limitations in triggering new arrhythmic pathologies. Traditional Chinese Medicine (TCM), with its extensive historical background, has demonstrated notable efficacy and relatively low incidence of adverse effects following millennia of clinical application [9, 10]. As an alternative therapeutic avenue, Chinese herbal medicine exhibits promise in modulating cardiac ion channels and exerting antiarrhythmic effects [11-15]. Additionally, systematic reviews and meta-analyses have underscored the advantages of combining acupuncture with the oral administration of Chinese herbal remedies for arrhythmia management [10]. Notably, TCM interventions have shown efficacy in arrhythmia treatment by ameliorating vascular endothelial function and providing anti-inflammatory therapy [16, 17]. GGLM, a renowned traditional Chinese herbal formula, traces its origins to Zhang Zhongjing's ” Treatise on Cold-Induced Febrile Diseases” during the late Eastern Han Dynasty in ancient China. Comprising four key herbs, Guizhi (Cassia twig), Gancao (Licorice), Muli (Ostreae Concha), and Longgu (Dragon bones), GGLM has garnered acclaim within TCM circles as a therapeutic analogue to “amiodarone” for arrhythmia. However, research confirming the correlation between the constituent components of GGLM and their metabolites within animal models and the pathogenic protein pathways implicated in arrhythmia remains lacking.

Network pharmacology integrates drug component targets and disease-related molecules into a biomolecular network. It employs GO enrichment analysis and KEGG enrichment analysis to predict the relationship between drugs and diseases. In essence, network pharmacology effectively elucidates the intricate therapeutic mechanisms of drugs, offering novel ideas and methodologies for interdisciplinary research bridging artificial intelligence and medicine. It also facilitates the analysis of extensive biomedical data and the transformation of data into actionable knowledge [18, 19]. Quantitative analysis of widely-targeted metabolome of plant metabolites using LC-MS/MS technology and high-sensitivity mass spectrometry is carried out to detect their major components [20]. UPLC-Q-TOF-MS/MS can be used for the detection of constituents in the drug-containing serum of rats after gavage of GGLM, which has the advantages of high sensitivity and high resolution, thus realizing the rapid isolation of a variety of complex constituents and the screening of potent constituents [21].

In earlier research, our team demonstrated the potential of Zhigancao decoction, a traditional Chinese medicine prescription, to mitigate atrial myocardial electrical and structural remodeling induced by rapid atrial pacing as a preventive measure against atrial fibrillation [22]. GGLM, another adjunct alternative therapy, is extensively utilized in China for managing patients with tachyarrhythmia. To further elucidate the mechanism of GGLM in treating tachyarrhythmia, this study will integrate network pharmacology, molecular docking, widely-targeted metabolite profiling, and untargeted metabolomics to identify the active components of GGLM, its key targets, and associated signaling pathways. Additionally, by establishing an animal model of tachyarrhythmia and administering GGLM intervention, we aim to validate its impact on relevant pathogenic proteins and signaling pathways. This research endeavors to offer theoretical support for the clinical application of GGLM.

## 2 MATERIALS AND METHODS

### 2.1 Network Pharmacological Analysis

#### 2.1.1 Chemical Composition Screening and Target Collection Analysis of GGLM

The chemical constituents of GGLM were investigated using the Traditional Chinese Medicine Systems Pharmacology Database (TCMSP) (https://old.tcmsp-e.com/tcmsp.php). This study focused on four herbs: Cassia twig, Licorice, Ostreae Concha, and Dragonsbones. To identify the active ingredients, compounds with an oral bioavailability (OB) ≥30% and drug-likeness (DL) ≥0.18 were selected based on the pharmacokinetics of TCM, including absorption, distribution, metabolism, and excretion. The UniProt database (https://www.uniprot.org/) was employed in TCMSP to standardize protein targets for these active ingredients. As Ostreae Concha and Dragons bones were not included in TCMSP, BATMAN-TCM (http://bionet.ncpsb.org.cn/batman-tcm/) was utilized to retrieve the chemical components and associated target genes related to Ostreae Concha and Dragons bones from TCM compounds using these terms as key search criteria.

#### 2.1.2 Acquisition of Arrhythmia Disease Targets

In the GeneCards, Online Mendelian Inheritance in Man (OMIM), and DisGeNET disease target databases, we searched for cardiac arrhythmia by entering the name of the disease “cardiac arrhythmia”. For searching, the acquired disease gene targets were summarized into one, the duplicate data were removed, and the unique values were retained. In this way, the target data of the disease were obtained. A Venn diagram was drawn using the Image GP tool to integrate the common targets of GGLM and disease.

#### 2.1.3 Construction of Network for Chinese Herbal Medicine - Active Ingredients - Intersection Targets

The information on traditional Chinese medicine, active ingredients, and intersecting targets was copied and organized into an Excel table and imported into Cytoscape software to construct the traditional Chinese medicine-active ingredient-intersecting target network diagram, visualize and analyze the relationship between traditional Chinese medicine-active ingredient-intersecting target, and explore the importance of active ingredients and related molecular mechanisms of action of GGLM in the treatment of cardiac arrhythmia.

#### 2.1.4 Protein-Protein Interaction Network (PPI)

The target genes shared by drugs and diseases were imported into the STRING database (https://cn.string-db.org/), and the protein-protein interaction (PPI) network diagram of potential targets of GGLM for arrhythmia treatment was obtained. Afterward, the TSV file was downloaded and imported into Cytoscape software for processing, and the PPI network diagram was drawn in network style, with the size and gradient color of nodes indicating the change in degree value.

#### 2.1.5 Gene Ontology (GO) and Pathway Analysis

The drug-disease common targets were entered into the Metascape database (https://metascape.org/), and the enrichment factor > 1.5, minimum count value of 3, and *P* less than 0.01 were set as the screening criteria for Gene Ontology (GO) and Kyoto Encyclopedia of Genes and Genomes (KEGG) enrichment analyses. Moreover, the top-ranked gene functions and pathways were plotted as bar charts and bubble diagrams.

### 2.2 Widely-Targeted Metabolomics Quantitative Analysis

#### 2.2.1 Drug Intervention

GGLM consists of four Chinese herbs, Cassia twig (15 g), Licorice (30 g), Ostreae Concha (30 g), and Dragon bones (30 g), purchased from the Hubei University of Chinese Medicine. According to our previous drug extraction method [8], all Chinese medicinal materials were soaked in 1 liter of pure water overnight, and impurities were removed after precipitation. Then, 1L of water was added and boiled at high temperature for 1 hour before impurities were filtered again. Afterward, 1L of water was added again for high-temperature frying. After boiling, the liquid was stirred continuously to prevent coagulation, and after heating for 30 minutes, the precipitation was filtered through the filter screen. Then, 0.945 g/mL of the filtered solution was concentrated in a water bath at 95°C. The resulting pharmaceutical solution was sterilized, sealed, and stored at 4°C and protected from light.

#### 2.2.2 Sample Preparation and Extraction

The GGLM was weighed at 150 mg and placed in a tissue dissolver. The sample was crushed at 50 Hz for 5 minutes. Oscillating GGLM scraps were vortexised (4°C, 3 times every 10 minutes), then incubated overnight at 4°C and centrifuged the next day (12000 rpm, 10 minutes). After centrifugation, 800μL supernatant was filtered by 0.22 μm filter membrane and analyzed by LC-MS.

#### 2.2.3 UPLC-MRM Quantification and Analysis

The resulting GGLM extracts were analyzed by using UPLC of QTRAP 6500 Plus (SCIEX, USA). The mass spectrometry data were viewed by the software analyst (SCIEX, USA), the metabolites were analyzed qualitatively and quantitatively by mass spectrometry, and the mass spectra were obtained by the extracted ion chromatograph (XIC, Extracted ion chromatograph). The horizontal coordinates are the residence time (min) of GGLM metabolites in RT, and the vertical coordinates are the ion flow intensity of target ion detection in GGLM metabolites. Each peak of a different color represents a detected metabolite. The characteristic ion of each substance was screened by a triple quadrupole, and the signal response intensity (CPS) of the characteristic ion was obtained using the mass spectrometry detector. The peak areas of the chromatographic peaks were calculated by integrating the downstream mass spectrometry data with the MultiQuant software, and the peak area of each peak represented the relative amount of the corresponding substance, and the peak areas of all the chromatographic peaks were exported for the subsequent statistical analyses. The mass spectrometric analysis of the compounds was carried out to confirm the chemical composition and structure of the compounds, to deduce the mass spectrometric cleavage pattern of the compounds, and to summarize the compounds according to their structural types.

### 2.3 Untargeted Metabolomics

#### 2.3.1 Preparation of Serum Samples

Twenty Sprague-Dawley (SD) rats of SPF grade were purchased from Liaoning Changsheng Biotechnology Co., Ltd. (Animal License: SCXK (Yu) 2020-0005). They were housed in the SPF Experimental Animal Center of Hubei University of Chinese Medicine (Experimental Unit License: SYXK (E) 2017-0067), ensuring constant temperature, humidity, and good ventilation. After acclimatization to feeding for one week, formal experiments were conducted. Twenty rats were randomly divided into 2 groups: control group (n=10) and drug group (n=10). The drug group received a dosage of 9.45 g/kg/day, administered once daily for 15 consecutive days, while the control group received an equivalent volume of water. After the final administration, anesthesia was induced by intraperitoneal injection of 3% sodium pentobarbital solution. Blood was collected from the abdominal aorta, left at room temperature for 30 minutes, and then centrifuged (12000 rpm, 15 min). The upper serum was stored at -80°C for non-targeted metabolomics analysis. All procedures were conducted to ensure the absence of pain in the animals.

#### 2.3.2 Metabolite Extraction

The preserved serum samples were melted until no ice was visible in the samples, 100 µL of each sample was added to an EP tube, 700 µL of extractant (methanol:acetonitrile: water=4:2:1, v/v/v) containing internal standard 1 was added to the tube, shaken for 1 min, and placed in the fridge at -20°C for 2h. Afterward, the samples were centrifuged for 15 minutes. After centrifugation, the supernatant was added to an equal volume of water and methanol, and the vortex oscillated evenly until all the samples were dissolved in the complex solution. Then, 180µL of methanol: water (1:1 v/v) was added, vortexed, and shaken for 10 min until all of the supernatant was dissolved in the compound solution, followed by centrifugation for another 15 min. Then, 20 µL of each sample was taken, mixed into a QC sample, and the prepared supernatant was taken for the test.

#### 2.3.3 UPLC-MS Analysis

A Waters UPLC I-Class Plus (Waters, USA) in tandem with a Q Exactive high-resolution mass spectrometer (Thermo Fisher Scientific, USA) was used for the separation and detection of metabolites. Mass spectrometry data were analyzed using Compound Discoverer 3.3 (Thermo Fisher Scientific, USA) software to obtain a data matrix containing metabolite peak area. The obtained data were used for further analysis and information processing. The BGI Metabolome Database and mzCloud databases were used for data comparison analysis (https://biosys.bgi.com/).

#### 2.3.4 Data Preprocessing and Holistic Analysis

Firstly, the data were normalized by probability quotient, and the relative peak area was calculated. Quality control-based robust LOESS was used to correct the batch effect, and then the metabolites with relative peak area variation coefficient greater than 30% were removed. Classification and functional annotation of the identified metabolites were performed to understand the classification status and functional properties of different metabolites. Functional annotation of the pathway was performed by the KEGG pathway database (https://www.genome.jp/kegg/pathway.html) to identify the major biochemical metabolic pathways and signaling pathways involved in the metabolites.

### 2.4 Molecular Docking

In order to validate the conclusions from the combined analysis of network pharmacology, quantitative analysis of widely-targeted metabolism, and untargeted metabolomics, we used the MAPK targets screened by network pharmacology and untargeted metabolomics, as the docking receptors, and the main active ingredients, calycosin, licochalcone B, and glabridin, which were quantitatively analyzed by widely-targeted metabolomics, as molecular docking ligands, and naringenin, medicarpin, formononetin, quercetin, isorhamnetin, and kaempferol as docked ligands for molecular docking. The 3D protein structures of the core targets were downloaded from the PDB database (https://www.rcsb.org/), and then the protein structures of the core targets were subjected to de-watering and de-liganding operations using Pymol 2.3.0 software. The structure of the core component protein was retrieved in sdf format by Pubchem (https://pubchem.ncbi.nlm.nih.gov/), and the sdf format was converted to pdf format by Open Babel 3.1.1. AutoDockTools 1.5.7 software was used to hydrogenate the processed protein structure of the core target and core component, and then AutoDock Vina 1.1.2 was used to run the script for the calculation of molecular docking and binding energies. All results were visualized using Pymol software.

### 2.5 Animal Experimentation

#### 2.5.1 Animal Grouping and Treatment

Thirty SD rats were randomly divided into 3 groups: control group (n=10), model group (n=10), and drug group (n=10). The drug group received daily oral administration of GGLM at a dosage of 9.45g/kg/d for 14 consecutive days. The model group and the control group received equivalent volumes of physiological saline by gavage. Starting from the seventh day after administration, the model group and the drug group were induced with isoproterenol (ISO) to create a model of rapid arrhythmia (5mg/kg, subcutaneous injection) for 7 consecutive days [23]. After the final administration, anesthesia was induced by intraperitoneal injection of 3% sodium pentobarbital solution. The electrocardiogram (ECG) of each group of rats was monitored using a small animal ECG machine, with baseline ECG recorded for 10 minutes. Subsequently, the hearts of the rats were carefully removed, rinsed with PBS at 4°C, and stored at -80°C for further use. All procedures were conducted to ensure the absence of pain in the animals.

#### 2.5.2 qT-PCR and Western blot

The mRNA expression levels of p38 MAPK, JNK, ERK1, NF-kb, IL-1β, and IL-12 in myocardial tissue were detected by real-time quantitative PCR (RT-qPCR). Total RNA was extracted using a Trizol reagent (Thermo, USA). Its concentration was determined and then reversely transcribed into cDNA. Then, the reaction amplification system was added and placed into a 7500 RT-qPCR instrument (Applied Biosystems, USA). PCR reaction conditions were 50°C for two minutes and 95°C for ten minutes, followed by 40 cycles of 95°C for 30s and 60°C for 30s. After the reaction, the relative quantitative analysis was performed with 2^-ΔΔct^ for RT-qPCR results.

Western blot was used to detect p38 MAPK, JNK, ERK1, NF-kb, IL-1β, and IL-12 in the myocardial tissue. For this purpose, 50mg of rat myocardial tissue was taken, and a RIPA lysis buffer was added to extract proteins. The protein concentration was determined using the BCA assay method. After determining the protein concentration, the protein samples were loaded and transferred onto membranes, followed by blocking. Primary antibodies against p38 MAPK, JNK, ERK1, NF-kb, IL-1β, and IL-12 were added at a dilution of 1:1000 and incubated overnight at 4°C. After washing, secondary antibodies corresponding to the respective primary antibodies were added at a dilution of 1:5000. Following further washing, the membranes were developed using an ECL substrate, and the bands were imaged and measured using an imaging system. The integrated optical density was analyzed and calculated using Image Pro Plus 5.0 software. The relative expression levels of each protein were calculated by comparing the band intensities with the grayscale values of the internal control GAPDH.

### 2.6 Statistical Analysis

All data were expressed as x̄±s and processed using SPSS 22.0 statistical software. One-way analysis of variance (ANOVA) was employed for multiple group comparisons. The LSD method was used for further pairwise comparison if the variance was homogeneous. Dunnett's T3 method was used for further pairwise comparison if the variance was not homogeneous. Differences were considered statistically significant at *P*<0.05.

## 3 RESULTS

### 3.1 Network Pharmacology Results

**Fig. (1):**
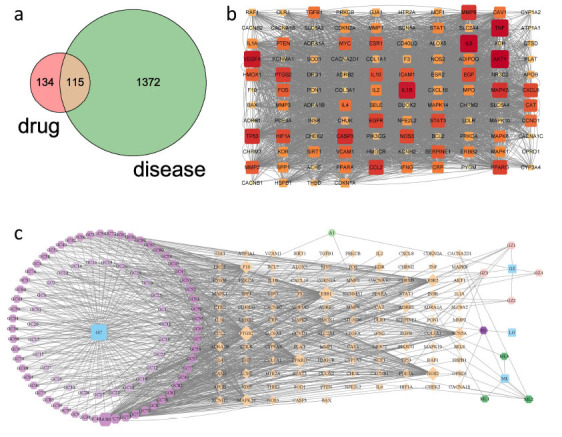
Key ingredients and key targets. (**a**) Venn analysis of GGLM and arrhythmia targets. (**b**) PPI network diagram of key targets for GGLM treatment of arrhythmia. (**c**) Network diagram of GGLM, active ingredients, and intersection targets.

The screening process involved assessing the active ingredients obtained, eliminating those lacking corresponding target interactions, resulting in a final set of 99 active ingredients for GGLM. This screening identified a total of 249 active ingredients of GGLM interacting with specific targets, along with 1,487 target genes associated with arrhythmia disorders. Among these, 115 target genes were common between GGLM and arrhythmia (Fig. **1a**). A network analysis graph of the TCM-Active Ingredients-Intersection Targets was constructed (Fig. **1b**) and sorted based on degree values. Notably, compounds, such as quercetin, kaempferol, and medicarpin, emerged as the top-ranking compounds. Subsequently, the shared target genes between the drugs and diseases were input into the STRING database to generate a protein-protein interaction (PPI) network graph (Fig. **1c**). Prominent genes, such as AKT1, IL6, TNF, IL1B, VEGFA, CASP3, TP53, MAPK3, MMP9, CCL2, PTGS2, and EGFR, occupied significant positions within the network.

**Fig. (2):**
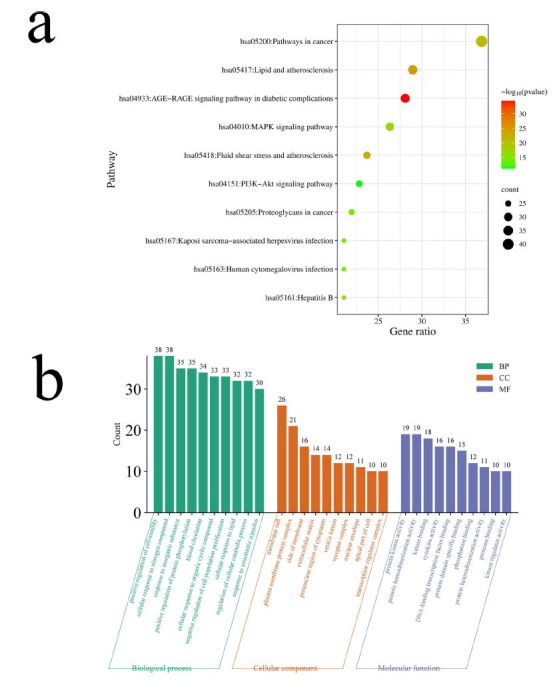
GO enrichment and KEGG analysis. (**a**) Histogram of BP, CC, and MF (Results for GO Enrichment Analysis). (**b**) Bubble diagram of KEGG pathway enrichment analysis.

To elucidate the potential mechanisms of GGLM in treating arrhythmia, it is essential to perform GO enrichment analysis and KEGG pathway analysis on the intersecting target genes (Fig. **2a**). In the GO enrichment analysis of biological processes, 4,934 entries were identified, encompassing diverse processes, including positive regulation of cell motility, cellular response to nitrogen compound, response to inorganic substance, positive regulation of protein phosphorylation, cellular response to organic cyclic compound, negative regulation of cell population proliferation, cellular response to lipid, and regulation of cellular catabolic process, among others. The molecular function analysis revealed 66 relevant entries, including protein kinase activity, protein homodimerization activity, kinase binding, cytokine activity, DNA-binding transcription factor binding, protein domain-specific binding, phosphatase binding, protein heterodimerization activity, and protease binding. In terms of cellular components, 421 relevant entries were obtained, encompassing components like membrane raft, plasma membrane protein complex, side of membrane, extracellular matrix, perinuclear region of cytoplasm, vesicle lumen, receptor complex, nuclear envelope, and transcription regulator complex.

The KEGG pathway enrichment analysis of GGLM treatment for cardiac arrhythmias identified a total of 244 relevant signaling pathways. Among these pathways, key signaling pathways associated with GGLM treatment for cardiac arrhythmias included pathways in cancer, lipid, and atherosclerosis, advanced glycation end product-receptor of advanced glycation end products (AGE-RAGE) signaling pathway in diabetic complications, MAPK signaling pathway, fluid shear stress and atherosclerosis, phosphatidylinositol-3-kinase-protein kinase B (PI3K/AKT) signaling pathway, and proteoglycans in cancer (Fig. **2b**).

### 3.2 Quantitative Analysis Results of Widely-Targeted Metabolism in Plants

**Fig. (3):**
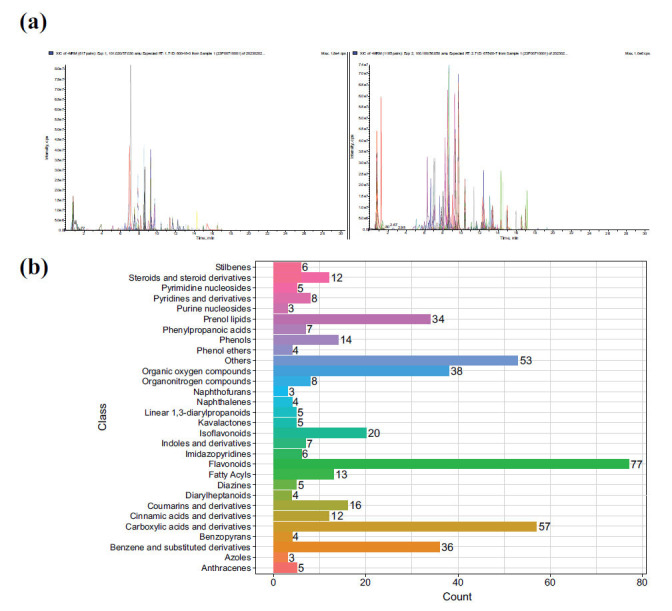
Widely-targeted metabolomics diagrams. (**a**) Positive and negative ion diagram. (**b**) Summary chart of metabolite classification information.

Through the comparison of positive and negative ion modes (Fig. **3a**), GGLT identified a total of 448 compounds (Supplement **S1**). These identified metabolites were classified and summarized (Fig. **3b**). The classification included 77 flavonoids, 57 carboxylic acids and derivatives, 38 organooxygen compounds, 36 benzene and substituted derivatives, 34 prenol lipids, 20 isoflavonoids, 16 coumarins and derivatives, 14 phenols, 13 fatty acyls, and 12 cinnamic acids and derivatives, as well as steroids and steroid derivatives.

### 3.3 Untargeted Metabolomics Results

**Fig. (4):**
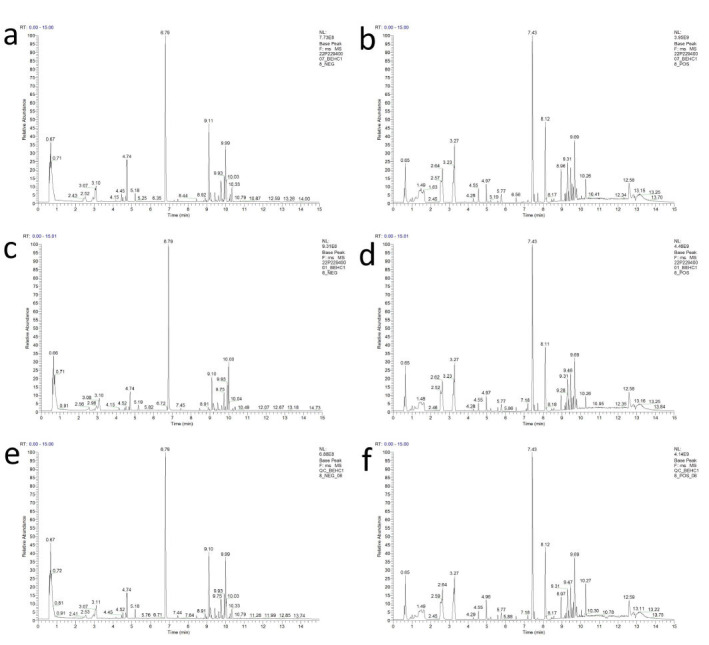
Positive and negative ion diagram. (**a**) Positive ionogram of the control group. (**b**) Negative ionogram of the control group. (**c**) Positive ionogram of the drug group. (**d**) Negative ionogram of the drug group. (**e**) Positive ionogram of the QC group. (**f**) Negative ionogram of the QC group.

By comparing the total ion chromatograms obtained from plasma samples of rats in the drug group, control group, and quality control (QC) samples using UPLC-MS/MS (Fig. **4**), a total of 3888 compounds were detected, with 1279 compounds being successfully identified.

**Fig. (5):**
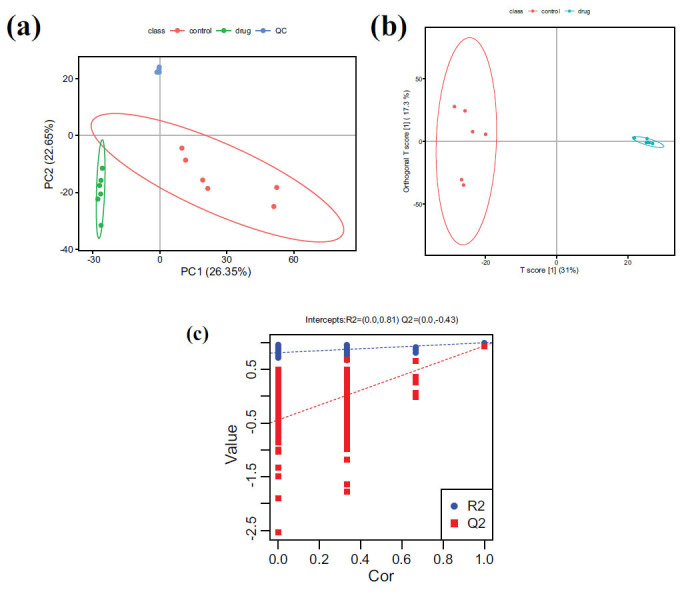
Multivariate statistical analysis of the plasma metabolites. (**a**) PCA Score graph of all samples. (**b**) Score diagram of OPLS-DA analysis mode. (**c**) Replacement test chart of OPLS-DA analysis model.

We utilized the LC-MS metabolomics analysis platform and conducted PCA and PLS-DA multivariate statistical analysis on the samples in positive and negative ion modes (Fig. **5**). In the PCA plot, the QC samples formed tight clusters, indicating the stability of the entire LC-MS analysis process with minimal system errors and excellent reproducibility, signifying good data quality. Additionally, we observed a distinct separation trend between the drug group and the control group in the PCA plot, suggesting differential metabolic features of endogenous metabolites in rats following drug administration. To further identify the differentially expressed metabolites in rats post-drug administration, the OPLS-DA plot exhibited significant separation between the drug group and the control group. To evaluate the model quality and prevent overfitting, we conducted 200 permutation tests on the OPLS-DA model. In each permutation, the randomly arranged R2 and Q2 values on the left side were all smaller than those on the right side (R2 Y=0.992, Q2=-0.43), indicating good predictive ability and no overfitting.

**Fig. (6):**
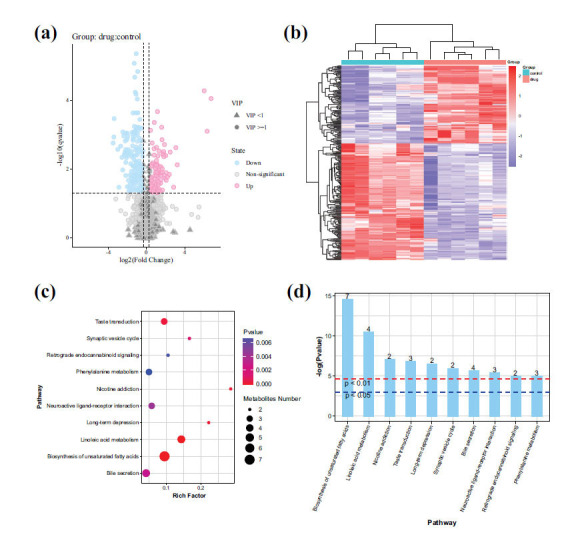
Differential metabolite analysis. (**a**) Volcano plot of differential metabolites. (**b**) Clustering on differential metabolites. (**c**) Bubble chart of metabolic pathway enrichment analysis. (**d**) Bar chart of pathway enrichment analysis.

We plotted a volcano plot of differentially expressed metabolites using the criteria of VIP ≥ 1, fold change ≥ 1.2 or ≤ 0.83, and q-value < 0.05 (Fig. **6a**). Each point in the volcano plot represents a variable, with the log2-transformed fold change on the x-axis and the -log10 transformed q-value on the y-axis. Blue points represent significantly downregulated metabolites, red points represent significantly upregulated metabolites, circles represent metabolites with VIP ≥ 1, triangles represent metabolites with VIP < 1, and non-significant metabolites are shown in gray. Using these criteria, we identified a total of 279 differentially expressed metabolites, with 112 upregulated and 167 downregulated (Supplement **2**).

In order to investigate the metabolic differences between the drug group and the control group, we performed normalization on 279 significantly different variables obtained from the two sample groups and generated a clustered heatmap (Fig. **6b**). The heatmap represents the variations in metabolite levels between the groups, where different colors indicate different relative abundance levels, ranging from blue to red, representing increasing expression. Upon observation, significant color differences were observed between the drug group and the control group in terms of metabolite levels. Specifically, compared to the control group, the drug group exhibited upregulation in 112 differential variables, accounting for 40.14% of all differential variables. These upregulated metabolites include Boldenone undecylenate, 4'-methoxy-3-morpholino-propiophenone,(2r)-3-({(2s)-2,3-dihydroxypro poxyphosphoryl}oxy)-2-hydroxypropyl (11z)-11-icosenoate, 18-β-glycyrrhetinic acid, Sm9625000, (1r,2r,3r,5z,7e)-2-(2-hydroxyethoxy)-9,10-secocholesta-5,7,10-triene-1,3,25-triol, *etc*. Furthermore, there were 167 downregulated differential variables in the drug group compared to the control group, which accounted for 59.86% of all differential variables. These downregulated metabolites included pruvanserin, protoporphyrin ix, (+/-)11(12)-dihet, R.g.-keto iii, lamtidine, (dl)-3-o-methyldopa, *etc*.

A total of 279 differential variables were classified according to their structures, resulting in 2 alkaloids, 23 benzenoids, 29 organoheterocyclic compounds, 32 organic acids and derivatives, 6 phenylpropanoids and polyketides, 2 fatty acids, 51 lipids and lipid-like molecules, 1 flavonoid, 1 venom, 1 fungal toxin, 13 organic nitrogen compounds and homogeneous non-metals, 1 compound, 3 terpenoids, 1 nucleoside, 1 nucleotide, and 1 analogue, and 113 other categories.

Enrichment analysis was performed on the 279 differential metabolites from the drug and control samples using the KEGG database (Fig. **6c** and **6d**). In the bubble plot, the larger and redder circles represent the major metabolic pathways associated with the differential metabolites. These pathways included biosynthesis of unsaturated fatty acids, linoleic acid metabolism, taste transduction, bile secretion, neuroactive ligand-receptor interaction, long-term depression, nicotine addiction, phenylalanine metabolism, retrograde endocannabinoid signaling, and synaptic vesicle cycle.

### 3.4 Comprehensive Analysis

**Fig. (7):**
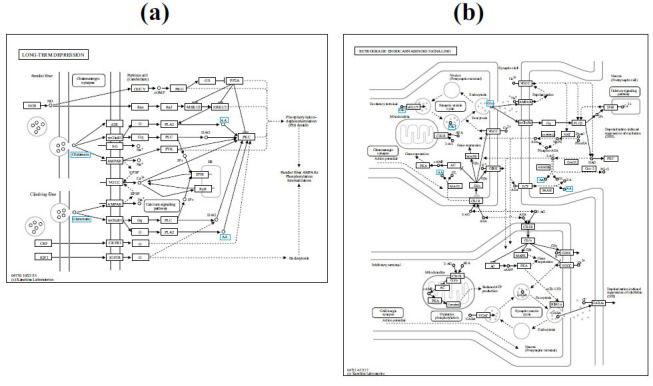
KEGG metabolic enrichment pathway. (**a**) Long-term depression. (**b**) Retrograde endocannabinoid.

To comprehensively and systematically understand the interrelationships among different pathways, upstream targets, and end metabolites, we integrated metabolomics and network pharmacology approaches to elucidate the mechanism of GGLM in treating cardiac arrhythmias in a more comprehensive, systematic, and precise manner. By combining the signaling pathways identified through network pharmacology with the major metabolic pathways identified through non-targeted metabolomics based on differential metabolite screening, we discovered two relevant signaling pathways: long-term depression and retrograde endocannabinoid signaling. Subsequently, these pathways were functionally annotated using the KEGG pathway database to determine the primary biochemical metabolic pathways, signal transduction pathways, and targets in which the metabolites participate (Fig. **7**). Furthermore, by integrating network pharmacology with the targets of differential metabolites, we identified five core targets: MAPK1, MAPK3, MAPK8, MAPK10, and MAPK14, along with two affected metabolites, L-glutamic acid, and arachidonic acid. Therefore, it is inferred that the mechanism underlying the therapeutic effect of GGLM on cardiac arrhythmias is primarily associated with the regulation of the MAPK pathway.

Based on the results of network pharmacology and targeted metabolomics quantification analysis, key active ingredients related to MAPK targets were identified and sorted by their relative abundances. The main active ingredients, in descending order of relative abundance, include calycosin, licochalcone B, glabridin, naringenin, medicarpin, formononetin, quercetin, isorhamnetin, and kaempferol (Table **1**). Therefore, we can infer that the therapeutic effect of Guizhi Gancao Longgu Muli Decoction in treating cardiac arrhythmias is primarily achieved through the regulation of the MAPK signaling pathway by active ingredients, such as calycosin, licochalcone B, glycerol, glabridin, naringenin, and medicarpin.

### 3.5 Molecular Docking Results

**Fig. (8):**
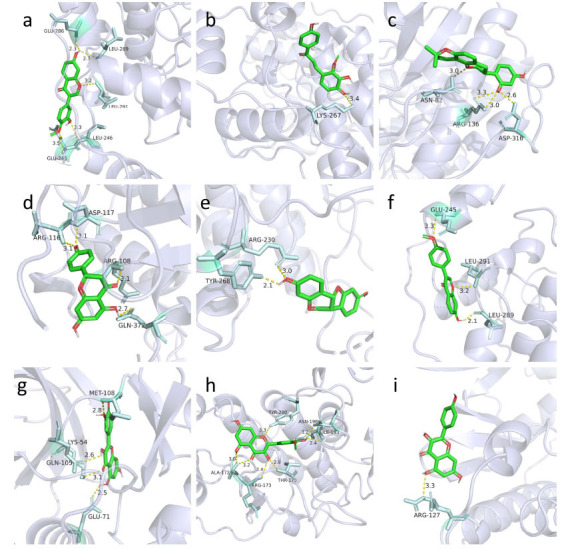
Molecular docking interaction diagrams. (**a**) The docking structure of calycosin with MAPK14. (**b**) The docking structure of licochalcone B with MAPK14. (**c**) The docking structure of glabridin with MAPK14. (**d**) The docking structure of naringenin with MAPK3. (**e**) The docking structure of medicarpin with MAPK10. (**f**) The docking structure of formononetin with MAPK14. (**g**) The docking structure of quercetin with MAPK1. (**h**) The docking structure of isorhamnetin with MAPK14. (**i**) The docking structure of kaempferol with MAPK8.

Molecular docking was conducted to explore the binding interactions between the core constituents of GGLM and the core target proteins. The binding affinities were calculated as binding energies, and the results were summarized (Table **2**). Additionally, the conformation with the lowest binding energy of the interaction was visualized. As shown in the figure, proteins and components were linked by hydrogen bonds, and the distance between these bonds could reflect the energy of the interaction. A shorter hydrogen bond indicates higher binding energy and a more stable conformation (Fig. **8**). A negative binding energy indicates binding activity, and a value below -5.0 kcal·mol-1 suggests strong binding affinity [24]. Furthermore, a lower binding energy reflects a more stable binding conformation between the constituents and target proteins. Molecular docking analysis revealed that the binding energies of nine core components and five target proteins were all below -5.0 kcal·mol^-1^, indicating strong binding activity. Specifically, the binding energies of calycosin, glabridin, formononetin, and isorhamnetin with MAPK14 (1A9U) were less than -7.0 kcal·mol^-1^. Similarly, the binding energies of naringenin and quercetin with MAPK3 (2ZOQ) and quercetin with MAPK1 (7E75) were below -7.0 kcal·mol^-1^. Additionally, the binding energy of kaempferol with MAPK8 (3ELJ) was also less than -7.0 kcal·mol^-1^, indicating robust binding activity and stable binding conformations. Notably, the binding energy between glabridin and MAPK14 (1A9U) was the lowest. In the visualization, amino acid residues ASN-82, ARG-136, and ASP-316 on MAPK14 (1A9U) formed four hydrogen bonds with glabridin, indicating a highly stable conformation. These findings align with predictions from network pharmacology and non-targeted metabolomics.

### 3.6 Experimental Results

The ECG results revealed a significant increase in heart rate among rats in the model group compared to those in the control group. Conversely, rats in the drug group exhibited a notable reduction in heart rate following GGLM administration compared to those in the model group, as depicted in Fig. (**9a**). Additionally, qRT-PCR results demonstrated a substantial upregulation in mRNA and protein expressions of p38 MAPK, JNK, ERK, NF-kB, IL-1β, and IL-12 in myocardial tissue of the model group compared to the control group (*P*<0.01). Subsequent GGLM administration intervention resulted in a significant decrease in the expression levels of these proteins and mRNA (*P* < 0.01), as shown in Fig. (**9b**-**d**).

## 4 DISCUSSION

Arrhythmias, a common and prevalent cardiovascular disease, refers to abnormalities in the heart's rhythm. Currently, the main treatment options for arrhythmias are medication therapy and radiofrequency ablation surgery. However, both approaches inevitably carry risks of surgery and adverse drug reactions. Therefore, there is an urgent need to explore new treatment options for arrhythmias. Although the therapeutic effect of GGLM on arrhythmias has been confirmed in China, the active ingredients and molecular mechanisms of GGLM have not been elucidated yet. In the past, it was believed that the remodeling of electric and structural properties of the heart and the formation of reentrant circuits were the fundamental mechanisms triggering and maintaining arrhythmias [25-27]. However, recent studies have indicated that the occurrence of arrhythmias is closely associated with endothelial dysfunction and inflammation in blood vessels [28-30]. Inflammation significantly contributes to the onset and progression of arrhythmias. Research has shown that, during the inflammatory response, IL-1β binding to its receptor can induce myocardial cells to produce substantial reactive oxygen species (ROS) [31-33]. This process activates downstream protein kinase Cε (PKCε), leading to the phosphorylation of serine-threonine residues on L-type calcium channels, which reduces their opening rate and increases the likelihood of arrhythmias. Additionally, IL-1β can trigger oxidation and phosphorylation of calcium/calmodulin-dependent protein kinase II (CaMKII), thereby enhancing the frequency of spontaneous contraction events in myocardial cells [34]. Furthermore, ROS activation of downstream NF-κB signaling pathways increases the secretion of inflammatory cytokines, such as TNF-α and IL-1β, initiating myocardial cell apoptosis, fibroblast proliferation and differentiation, and extracellular matrix secretion. This cascade ultimately results in atrial tissue fibrosis, structural and functional abnormalities in myocardial cells, and the onset of arrhythmias [35, 36]. The inflammatory cytokine TNF-α can affect the stability of myocardial action potentials by influencing intercellular conduction between myocardial cells, leading to arrhythmias [37, 38]. TNF-α can also increase the secretion of matrix metalloproteinase-2 (MMP-2) and MMP-9 through the activation of the transforming growth factor-β (TGF-β) pathway, further promoting the synthesis and secretion of atrial collagen, leading to atrial fibrosis and structural remodeling. These changes ultimately contribute to the occurrence of arrhythmias [39-41].

Combining the results of network pharmacology, widely targeted metabolomics detection, and non-targeted metabolite detection, we have confirmed the significant role of the MAPK signaling pathway in the process of GGLM treating arrhythmias. It mediates the proliferation, differentiation, apoptosis, and inflammatory responses of cardiovascular cells, thereby influencing the occurrence and development of arrhythmias [42-44]. The MAPK signaling pathway is also a crucial signaling pathway in the body's response to various stimuli, closely associated with endothelial dysfunction, with p38 MAPK playing a key role in this pathway [45, 46]. Moreover, the MAPK signaling pathway serves as a pro-inflammatory system, and its excessive activation is associated with various inflammatory diseases [47-50]. Inhibiting the MAPK signaling pathway can reduce the expression of inflammatory cytokines, such as IL-1β, IL-6, and IL-12, thereby suppressing inflammation in myocardial tissue [51, 52].

The clinical efficacy of GGLM in treating arrhythmias has been confirmed in China, with high safety and no apparent toxic side effects. GGLM is composed of Cassia twig, licorice, Ostreae Concha, and Dragon bones. Among them, *licorice* has a clear antiarrhythmic effect, as demonstrated by animal experiments showing its ability to protect the heart and treat arrhythmias by modulating immunity and oxidation [53-55]. Furthermore, animal studies have reported that *licorice* can treat arrhythmias by regulating myocardial cell potassium and sodium ion channels [56-58].

However, due to the complex composition of Chinese medicine compounds and their advantages of exerting multi-component, multi-target, and multi-pathway effects, our widely-targeted metabolomics analysis identified 448 compounds in GGLM. These compounds include 77 flavonoids, 57 carboxylic acids and derivatives, 38 organic oxygen compounds, 36 benzene and substituted derivatives, 34 isoprenoid lipids, 20 isoflavones, 16 coumarins and derivatives, 14 phenols, 13 fatty acyls, and 12 cinnamic acid and derivatives, as well as steroids and steroid derivatives. Among them, calycosin, licochalcone B, glabridin, naringenin, medicarpin, formononetin, quercetin, isorhamnetin, and kaempferol are the major constituents, with high contents. Calycosin, as one of the primary active ingredients in GGLM, has demonstrated various biological effects, including antioxidant, pro-angiogenic, and anti-inflammatory properties [59-62]. Licochalcone B, another major active ingredient, exhibits anti-inflammatory and anti-apoptotic effects on the heart [63-65]. Glabridin, a flavonoid compound, exerts anti-inflammatory, antioxidant, and anti-atherosclerotic effects [66, 67]. Naringenin, a flavonoid with potential cardioprotective effects, can protect ischemic myocardial cells by inhibiting the NLRP3 inflammasome pathway [68-70]. Additionally, the abundant presence of naringenin in GGLM can alleviate ISO-induced myocardial hypertrophy by modulating the AMPK/NOX2/MAPK signaling pathway [71-73]. Naringenin can also protect myocardial cells by exerting anti-inflammatory, antioxidant, and anti-apoptotic effects [74].

Through network pharmacology analysis, it was found that calycosin, licochalcone B, glabridin, naringenin, medicarpin, formononetin, quercetin, isorhamnetin, and kaempferol are closely related to the MAPK signaling pathway and interact with MAPK1, MAPK3, MAPK8, MAPK10, and MAPK14 targets. Subsequently, we used molecular docking to verify the binding activity and stability of conformations between the components and targets. The molecular docking results showed that the binding energies of the main active components in GGLM with the five target proteins were all below -5.0 kcal·mol-1, indicating good binding activity and stable conformation. These findings align with the predictions from network pharmacology and non-targeted metabolomics. Nevertheless, these results necessitate additional validation through *in vitro* animal experiments.

To validate our hypothesis further, we induced rapid arrhythmia in a rat model using isoproterenol. The ECG results aligned with our expectations. In the model group, rats displayed a notable elevation in heart rate. Notably, after intervention with GGLM, the rats' heart rates temporarily decreased to a level lower than that of the control group. Additionally, the protein and mRNA expression levels of p38 MAPK, JNK, ERK, NF-kB, IL-1β, and IL-12 in myocardial tissues of the GGLM-treated rats were significantly lower than those in the model group, providing further evidence that GGLM may mitigate inflammation and manage tachyarrhythmia by suppressing the MAPK signaling pathway.

## CONCLUSION

In summary, our study integrated network pharmacology, widely-targeted metabolomics quantitative analysis, non-targeted metabolomics, molecular docking, and animal experiments, confirming the potential efficacy of GGLM in addressing tachyarrhythmia. This effect is likely attributed to the presence of metabolites in GGLM containing numerous active ingredients, such as calycosin, licochalcone B, glabridin, naringenin, medicarpin, among others, which may ultimately exert therapeutic effects in rat models of arrhythmia by regulating the MAPK signaling pathway. Unfortunately, due to time and budget constraints, we were unable to assess the pathological changes in the heart tissues of rats. In future studies, we plan to further investigate the impact of GGLM on the pathological changes observed in cardiac tissue of rat models with arrhythmia. Our aim is to comprehensively evaluate the overall therapeutic efficacy of GGLM and provide additional, comprehensive evidence supporting the utilization of Chinese herbal medicine in arrhythmia treatment.

## Figures and Tables

**Fig. (9):**
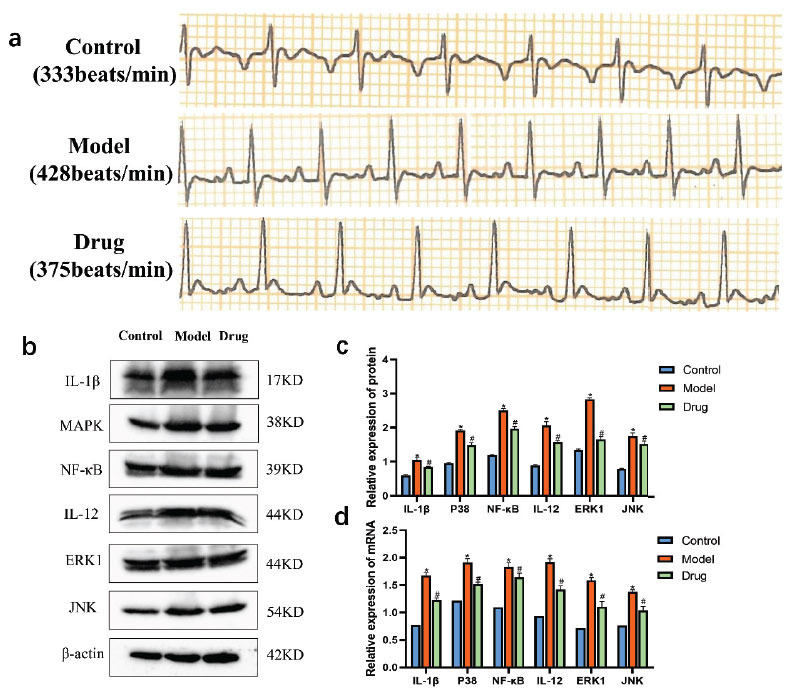
Effect of GGLM on tachyarrhythmia model rats. (**a**) Comparison of ECG among three groups of rats. (**b-c**) Protein expression levels of IL-1β, p38 MAPK, NF-kB, IL-12, ERK1, and JNK. (**d**) mRNA expression levels of IL-1β, p38 MAPK, NF-kB, IL-12, ERK1, and JNK. (Compared with the Control group,*P<0.05; Compared with the Model group, #*P*<0.05, N=3).

**Table 1:** Comprehensive analysis of network pharmacology and targeted metabolism.

	**Network Pharmacology Components**	**Protein Target**	**Targeted Metabolic Detection Components**
1.	Calycosin	MAPK14	Calycosin-7-O-β-D-glucoside
			Calycosin
2.	Licochalcone B	MAPK14	Licochalcone B
3.	Glabridin	MAPK14	Glabridin
4.	Naringenin	MAPK3	8-Prenylnaringenin
			Naringenin chalcone
			Naringenin
			Naringenin 7-O-glucoside (Prunin)
5.	Medicarpin	MAPK10	Medicarpin
6.	Formononetin	MAPK14	Formononetin
7.	Quercetin	MAPK1	Di-O-methylquercetin
			Quercetin 7-rhamnoside
8.	Isorhamnetin	MAPK14	Isorhamnetin-3-O-nehesperidine
			Isorhamnetin 3-O-glucoside
9.	Kaempferol	MAPK8	Kaempferol 3-O-galactoside (Trifolin)
			Kaempferol-7-O-β-D-glucopyranoside
			Kaempferol
			DIHYDROKAEMPFEROL

**Table 2:** Scoring of docking energy and docking parameters between key compounds and core target molecules.

	**Ingredients**	**Protein Target (PDB ID)**	**Binding Energy (kcal·mol^-1^)**
1.	Calycosin	MAPK14(1A9U)	-7.4
2.	Licochalcone B	MAPK14(1A9U)	-6.6
3.	Glabridin	MAPK14(1A9U)	-8.1
4.	Naringenin	MAPK3(2ZOQ)	-7.1
5.	Medicarpin	MAPK10(4KKG)	-6.2
6.	Formononetin	MAPK14(1A9U)	-7.1
7.	Quercetin	MAPK1(7E75)	-7.5
8.	Isorhamnetin	MAPK14(1A9U)	-7.0
9.	Kaempferol	MAPK8(3ELJ)	-7.1

## Data Availability

Not applicable.

## LIST OF ABBREVIATIONS

GGLM: Guigan Longmu Decoction
TCM: Traditional Chinese Medicine
UPLC-Q-TOF-MS/MS: Ultra-High-Performance Liquid Chromatography-Quadrupole-Time-Of-Flight Mass Spectrometry
MRM: Multiple Reaction Monitoring
TCMSP: Traditional Chinese Medicine Systems Pharmacology Database
BATMAN-TCM: The Bioinformatics Analysis Tool for Molecular Mechanism of Traditional Chinese Medicine
OB: Oral Bioavailability
GO: Gene Ontology
PPI: Protein-Protein Interaction
KEGG: Kyoto Encyclopedia of Genes and Genomes
RT: Retention Time
MAPK: Mitogen-Activated Protein Kinase
QC: Quality Control
BP: Biological Processes
MF: Molecular Function
CC: Cellular Components
ERK: Extracellular Signal-Regulated Protein Kinase
JNK: c-Jun N-Terminal Kinase
