# Supplementary material for: Mechanism of Guigan Longmu Decoction in the Treatment of Arrhythmias Based on Network Pharmacology and Untargeted Metabolomics Assays
Source: Comb Chem High Throughput Screen. 2024 Jun 3;28(8):1384–401. doi: 10.2174/0113862073293313240519161145 (PMC12376120; doi:10.2174/0113862073293313240519161145)
Supplement: Supplementary file 1 [file CCHTS-28-8-1384_SD1.pdf]

Supplementary Material

Mechanism of Guigan Longmu Decoction in the Treatment of Arrhythmias Based on Network Pharmacology and Untargeted Metabolomics Assays

Tuo Deng[1,#], Sheng Guo[2,#], Jie Yang[2], Xiao-yu Huang[1], Xiao-bin Lu [1], Jing Lu[1], Liang Liu[1,*] and Ze Du[1]

[1]Hubei Provincial Hospital of Traditional Chinese Medicine, Wuhan, Hubei, 430061, China; [2]Hubei University of Chinese Medicine, Wuhan, Hubei, 430065, China

Table S1.

BWID	23P00710001	23P00710002	23P00710003	Compound name	Molecular_Formula	Molecular_Weight	HMDB.ID	KEGG.ID	Super.class	Class	sub_class	Family	Pathway
551-15-5	663654912	635506432	680395200	Liquiritin 甘草苷	C21H22O9	418.39	HMDB0029520	C16989	Phenylpropanoids and polyketides	Flavonoids	Flavonoid glycosides	Flavonoids	NA
199796-12-8	363608320	330539648	332179520	Liguiritigenin-7-O-β-D-apiosyl-4'-O-β-D-glucoside 甘草苷元-7-O-β-D-芹糖-4'-O-β-D-葡萄糖苷	C26H30O13	550.509	NA	NA	NA	NA	NA	NA	NA
51059-44-0	316235776	304282976	346687872	Wogonoside 汉黄芩苷	C22H20O11	460.388	NA	NA	NA	NA	NA	NA	NA
36948-76-2	315825472	303858208	346181088	Oroxylin A-7-O-β-D-glucuronide 千层纸素A-7-0-β-D-葡萄糖醛酸苷	C22H20O11	460.388	NA	NA	NA	NA	NA	NA	NA
147-85-3	287337792	271977632	265519168	2-Pyrrolidinecarboxylic acid L-脯氨酸	C5H9NO2	115.131	HMDB0000162	C00148	Organic acids and derivatives	Carboxylic acids and derivatives	Amino acids, peptides, and analogues	Amino acids	NA
21967-41-9	264566352	281557056	312906880	Baicalin 黄芩苷	C21H18O11	446.361	HMDB0041832	C10025	Phenylpropanoids and polyketides	Macrolides and analogues	null	Flavonoids	NA
557-24-4	234912080	223161920	254116224	SR	C4H5NO3	115.087	NA	C01596	NA	NA	NA	NA	map00760 Nicotinate and nicotinamide metabolism; map01100 Metabolic pathways; map01120 Microbial metabolism in diverse environments
344-25-2	225493520	240451072	221380336	D-Proline	C5H9NO2	115.131	HMDB0003411	C00763	Organic acids and derivatives	Carboxylic acids and derivatives	Amino acids, peptides, and analogues	Amino acids, peptides, and analogues	NA
5041-81-6	199879696	177768080	171801824	Isoliquiritin 异甘草苷	C21H22O9	418.394	HMDB0037318	C16978	Phenylpropanoids and polyketides	Flavonoids	Flavonoid glycosides	Flavonoids	NA
57-50-1	174095488	199215424	150565312	Sucrose 蔗糖	C12H22O11	342.297	HMDB0000258	C00089	Organic oxygen compounds	Organooxygen compounds	Carbohydrates and carbohydrate conjugates	Carbohydrates	NA
528-50-7	147882320	165471152	158575952	D-(+)-Cellobiose	C12H22O11	342.297	HMDB0000055	NA	Organic oxygen compounds	Organooxygen compounds	Carbohydrates and carbohydrate conjugates	Carbohydrates	NA
471-87-4	145398576	152067328	163916064	Stachydrine 水苏碱	C7H13NO2	143.18	HMDB0004827	C10172	Organic acids and derivatives	Carboxylic acids and derivatives	Amino acids, peptides, and analogues	Amino acids, peptides, and analogues	NA
99-20-7	139120192	153381440	148128240	D-(+)-Trehalose	C12H22O11	342.297	HMDB0000975	C01083	Organic oxygen compounds	Organooxygen compounds	Carbohydrates and carbohydrate conjugates	Carbohydrates	NA
578-86-9	134968128	112990664	108388368	Liquiritigenin 甘草素	C15H12O4	256.253	HMDB0029519	C09762	Phenylpropanoids and	Flavonoids	Flavans	Flavonoids	NA

									polyketides				
535-83-1	130873392	122906816	97480200	Trigonelline	C7H7NO2	137.136	HMDB0000875	C01004	Alkaloids and derivatives	null	null	Alkaloids and derivatives	NA
486-62-4	115047336	120777920	125940824	Ononin 芒柄花苷	C22H22O9	430.405	HMDB0033987	C10509	Phenylpropanoids and polyketides	Isoflavonoids	Isoflavonoid O-glycosides	Flavonoids	NA
13241-33-3	109738616	90012480	111470968	Neohesperidin 新橙皮苷	C28H34O15	610.561	HMDB0030748	C09806	Phenylpropanoids and polyketides	Flavonoids	Flavonoid glycosides	Flavonoids	NA
520-26-3	97451288	107441304	99715368	Hesperidin 橙皮苷	C28H34O15	610.561	HMDB0003265	C09755	Phenylpropanoids and polyketides	Flavonoids	Flavonoid glycosides	Flavonoids	NA
91-64-5	96938456	105326248	127155760	Coumarin 香豆素	C9H6O2	146.143	HMDB0001218	C05851	Phenylpropanoids and polyketides	Coumarins and derivatives	null	Coumarins and derivatives	NA
6138-41-6	95188360	79226864	89669736	Trigonelline HCl 盐酸胡芦巴碱	C7H8ClNO2	173.597	NA	NA	NA	NA	NA	NA	NA
20633-67-4	91869472	78209912	84528984	Calycosin-7-O-β-D-glucoside 毛蕊异黄酮苷	C22H22O10	446.404	NA	NA	NA	NA	NA	NA	NA
961-29-5	87443584	80319392	83804544	Isoliquiritigenin 异甘草素	C15H12O4	256.253	HMDB0037316	C08650	Phenylpropanoids and polyketides	Linear 1,3-diarylpropanoids	Chalcones and dihydrochalcones	Polyketides[PK]	NA
520-36-5	86395536	88060720	82943336	Apigenin 芹菜素	C15H10O5	270.237	HMDB0002124	C01477	Phenylpropanoids and polyketides	Flavonoids	Flavones	Flavonoids	NA
945721-10-8	85632720	87687192	104631864	7-O-Ethylmorroniside 7-氧乙基莫诺苷	C19H30O11	434.435	NA	NA	NA	NA	NA	NA	NA
34221-41-5	83564768	78360448	71469792	Retrochalcone 刺甘草查尔酮	C16H14O4	270.28	NA	NA	NA	NA	NA	NA	NA
36052-37-6	82452480	76374480	77320176	Alpinetin 山姜素	C16H14O4	270.28	NA	NA	NA	NA	NA	NA	NA
1360-76-5	78342424	62051032	90831216	Ranaconitine 冉乌头碱	C32H44N2O9	600.7	NA	NA	NA	NA	NA	NA	NA
29741-09-1	72048192	88926624	107349560	Apigenin 7-O-glucuronide 芹菜素-7-O-葡萄糖醛酸苷	C21H18O11	446.361	NA	NA	NA	NA	NA	NA	NA
59914-91-9	71085656	68422120	79779616	Vicenin III 维采宁III	C26H28O14	564.492	NA	NA	NA	NA	NA	NA	NA
52012-29-0	69915832	65304160	73649952	Isoschaftoside 异夏佛塔苷	C26H28O14	564.492	NA	NA	NA	NA	NA	NA	NA
67909-49-3	68067864	60905596	64400352	Dehydroevodiamine 去氢吴茱萸碱	C19H15N3O	301.342	NA	NA	NA	NA	NA	NA	NA
79165-06-3	64758332	72880392	84144968	Diammonium glycyrrhizinate 甘草酸二铵	C42H68N2O16	856.993	NA	NA	NA	NA	NA	NA	NA
578-74-5	61664848	49503932	50518728	Apigenin-7-O-β-D-glucoside 芹菜素-7-O-β-D-吡喃葡萄糖苷	C21H20O10	432.378	HMDB0037340	C04608	Phenylpropanoids and polyketides	Flavonoids	Flavonoid glycosides	Flavonoids	NA
84272-85-5	61164872	53505852	59570412	5-O-Methylvisammioside 5-O-甲基维斯阿米醇苷	C22H28O10	452.452	NA	NA	NA	NA	NA	NA	NA
70872-29-6	60992052	55443900	57093192	Isoxanthohumol 异黄腐醇	C21H22O5	354.396	NA	NA	NA	NA	NA	NA	NA
58749-23-8	59371584	72692928	58575420	Licochalcone B 甘草查尔酮B	C16H14O5	286.279	HMDB0037320	NA	Phenylpropanoids and polyketides	Linear 1,3-diarylpropanoids	Chalcones and dihydrochalcones	Polyketides[PK]	NA
57396-78-8	55784412	61552644	57798224	Oroxin A 木蝴蝶苷A	C21H20O10	432.378	NA	NA	NA	NA	NA	NA	NA
14259-46-2	46728652	35607288	53586892	Narirutin 芸香柚皮苷	C27H32O14	580.535	HMDB0033740	C09793	Phenylpropanoids and polyketides	Flavonoids	Flavonoid glycosides	Flavonoids	NA
77-92-9	45504296	43007152	50865632	Citric acid 柠檬酸	C6H8O7	192.124	HMDB0000094	C00158	Organic acids and derivatives	Carboxylic acids and derivatives	Tricarboxylic acids and derivatives	Organic acids	NA
10236-47-2	44691152	34237712	50537672	Naringin 柚皮苷	C27H32O14	580.53	HMDB0002927	C09789	Phenylpropanoids and polyketides	Flavonoids	Flavonoid glycosides	Flavonoids	NA
28610-30-2	42979372	39636232	35916912	Isoanhydroicaritin 异脱水淫羊霍素	C21H20O6	368.38	NA	NA	NA	NA	NA	NA	NA

Glycyrol	39821820	35479720	38286716	Glabridin 光甘草定	C20H20O4	324.37	HMDB0034188	C10421	Phenylpropanoids and polyketides	Isoflavonoids	Pyranoisoflavonoids	Flavonoids	NA
19879-32-4	39048848	39522784	38818520	Bavachin 补骨脂甲素	C20H20O4	324.37	NA	NA	NA	NA	NA	NA	NA
40957-83-3	38328084	36917648	39263296	Glycitein 黄豆黄素	C16H12O5	284.263	HMDB0005781	C14536	Phenylpropanoids and polyketides	Isoflavonoids	Isoflav-2-enes	Flavonoids	NA
53846-50-7	34947696	25622022	29989486	8-Prenylnaringenin 8-异戊烯基柚皮素	C20H20O5	340.37	NA	NA	NA	NA	NA	NA	NA
493633	34812924	36189956	34090956	4-amino-butyricaci-methylester	C5H11NO2	117.146	NA	NA	NA	NA	NA	NA	NA
62949-79-5	33364524	30741762	30877626	Mulberrin 桑黄酮	C25H26O6	422.47	HMDB0029507	NA	Phenylpropanoids and polyketides	Flavonoids	Flavones	Flavonoids	NA
35354-74-6	30834176	29726932	29215580	Honokiol 和厚朴酚	C18H18O2	266.334	NA	NA	NA	NA	NA	NA	NA
73692-50-9	30051874	26931258	28441014	Naringenin chalcone 柚皮苷查尔酮	C15H12O5	272.253	NA	NA	NA	NA	NA	NA	NA
480-41-1	29919838	26861816	28208002	Naringenin 柚皮素	C15H12O5	272.253	HMDB0002670	C00509	Phenylpropanoids and polyketides	Flavonoids	Flavans	Flavonoids	NA
529-55-5	28468176	26410760	27128882	Naringenin 7-O-glucoside (Prunin)	C21H22O10	434.393	NA	C09099	NA	NA	NA	Flavonoids	map00941 Flavonoid biosynthesis
68-41-7	27170586	26185396	31206128	D-cycloserine	C3H6N2O2	102.092	HMDB0014405	C08057	Organoheterocyclic compounds	Azolines	Isoxazolines	Isoxazolines	NA
155-58-8	25790114	34201732	30272800	Rhaponticin 土大黄苷	C21H24O9	420.41	NA	C10288	NA	NA	NA	Polyketides[PK]	NULL
52328-97-9	24165212	21417980	20032076	Tetramethyl-curcumin 四甲基姜黄素	C25H28O6	424.486	NA	NA	NA	NA	NA	NA	NA
60-41-3	23881536	20210372	23802644	Strychnine sulfate 马钱子碱硫酸盐	C42H46N4O8S	766.902	NA	NA	NA	NA	NA	NA	NA
7533-40-6	23705516	29971346	25658876	L(+)-Leucinol L-亮氨醇	C6H15NO	117.189	NA	NA	NA	NA	NA	NA	NA
3681-93-4	23661592	17437744	12478084	Vitexin 牡荆素	C21H20O10	432.378	NA	C01460	NA	NA	NA	Flavonoids	map00941 Flavonoid biosynthesis; map00944 Flavone and flavonol biosynthesis
572-31-6	22990564	27457596	22320420	Engeletin 黄杞苷	C21H22O10	434.39	NA	NA	NA	NA	NA	NA	NA
107-43-7	21769020	28152814	23919146	Betaine 甜菜碱	C5H11NO2	117.146	NA	C00719	NA	NA	NA	Amino acids, peptides, and analogues	map00260 Glycine, serine and threonine metabolism; map01100 Metabolic pathways; map02010 ABC transporters
480-11-5	21334760	17807286	19885230	Oroxylin A 千层纸素A	C16H12O5	284.263	NA	NA	NA	NA	NA	NA	NA
632-85-9	20652848	18844356	16596456	Wogonin 汉黄芩素	C16H12O5	284.263	NA	C10197	NA	NA	NA	Flavonoids	NULL
491-80-5	20617514	18828768	16562328	Biochanin A 鹰嘴豆芽素A	C16H12O5	284.263	HMDB0002338	C00814	Phenylpropanoids and polyketides	Isoflavonoids	O-methylated isoflavonoids	Flavonoids	NA
26544-34-3	20135054	19217510	21486852	Apiin 芹菜苷	C26H28O14	564.492	HMDB0030843	C04858	Phenylpropanoids and polyketides	Flavonoids	Flavonoid glycosides	Flavonoids	NA
77-95-2	20007126	18024572	21257212	Quinic acid 右旋奎宁酸	C7H12O6	192.167	HMDB0003072	C00296	Organic oxygen compounds	Organooxygen compounds	Alcohols and polyols	Alcohols	NA
32383-76-9	18612808	17204390	16210444	Medicarpin 美迪紫檀素	C16H14O4	270.28	NA	C10503	NA	NA	NA	Flavonoids	map00943 Isoflavonoid biosynthesis; map01061 Biosynthesis of phenylpropanoids; map01110 Biosynthesis of secondary metabolites

524-17-4	18429710	16922612	21247902	Dauricine 蝙蝠葛碱	C38H44N2O6	624.766	NA	C09419	NA	NA	NA	Alkaloids	NULL
73069-14-4	18337550	27660184	23332200	Atractylenolide II 白朮内酯II	C15H20O2	232.318	NA	NA	NA	NA	NA	NA	NA
51938-32-0	16417993	18165124	17979882	Schaftoside 夏佛塔苷	C26H28O14	564.49	NA	C10181	NA	NA	NA	Flavonoids	NULL
114482-86-9	15647015	21311340	15357818	Oroxin B 木蝴蝶苷B	C27H30O15	594.518	NA	NA	NA	NA	NA	NA	NA
27661-51-4	15633930	16323829	12670115	Leucoside 山奈酚3-O-桑布双糖苷	C26H28O15	580.492	NA	NA	NA	NA	NA	NA	NA
50-81-7	15079466	10623023	10707627	Ascorbic acid	C6H8O6	176.124	HMDB0000044	C00072	Organoheterocyclic compounds	Dihydrofurans	Furanones	Furanones	NA
23313-21-5	14238209	14685268	12438252	Emodin-8-O-β-D-glucopyranoside 大黄素-8-O-β-D-吡喃葡萄糖苷	C21H20O10	432.378	NA	NA	NA	NA	NA	NA	NA
20243-59-8	14012825	12951436	13095067	Hydroxygenkwanin 羟基芫花素	C16H12O6	300.263	HMDB0037339	NA	Phenylpropanoids and polyketides	Flavonoids	O-methylated flavonoids	Flavonoids	NA
891986	12862811	13461035	16499893	Dacarbazine	C6H10N6O	182.183	HMDB0014989	C06936	Organic acids and derivatives	Carboxylic acids and derivatives	Carboxylic acid derivatives	Organic acids	NA
21913-99-5	12851272	13711686	14197344	Butin	C15H12O5	272.25	NA	NA	NA	NA	NA	NA	NA
475-67-2	12798435	14406206	15132606	S-Isocorydine(+) 异紫堇定碱	C20H23NO4	341.401	HMDB0030184	C09549	Alkaloids and derivatives	Aporphines	null	Alkaloids and derivatives	NA
1088-17-1	12154899	11858971	16174951	Isomeranzin 异橙皮内酯	C15H16O4	260.285	NA	NA	NA	NA	NA	NA	NA
69-72-7	12070009	10290908	13869374	Salicylic acid 水杨酸	C7H6O3	138.121	HMDB0001895	C00805	Benzenoids	Benzene and substituted derivatives	Benzoic acids and derivatives	Benzene and derivatives	NA
73-40-5	11640624	11688993	10775115	Guanine 鸟嘌呤	C5H5N5O	151.126	HMDB0000132	C00242	Organoheterocyclic compounds	Imidazopyrimidines	Purines and purine derivatives	Purines and derivatives	NA
529-59-9	11494267	11934092	9980517	Genistin 染料木苷	C21H20O10	432.378	HMDB0033988	C09126	Phenylpropanoids and polyketides	Isoflavonoids	Isoflavonoid O-glycosides	Flavonoids	NA
521-61-9	11271031	11775674	11900955	Emodin-3-methyl ether/Physcion 大黄素甲醚	C16H12O5	284.26	NA	C17045	NA	NA	NA	Quinone	NULL
16562-13-3	10795815	9981823	9821195	L-Stepholidine 左旋千金藤啶碱	C19H21NO4	327.374	NA	NA	NA	NA	NA	NA	NA
63038-10-8	10494657	9419984	11699689	Senkyunolide A 洋川芎内酯A	C12H16O2	192.254	NA	C17853	NA	NA	NA	NA	NULL
675-20-7	10309048	10019268	10297785	2-Piperidone	C5H9NO	99.131	HMDB0011749	NA	Organoheterocyclic compounds	Piperidines	Piperidinones	Piperidinones	NA
80554-58-1	10259116	9082365	8403821	Schinifoline 青花椒碱	C17H23NO	257.371	NA	NA	NA	NA	NA	NA	NA
152-95-4	10161023	11412865	10839653	Sophoricoside 槐角苷	C21H20O10	432.378	NA	NA	NA	NA	NA	NA	NA
23599-69-1	9871770	9576093	9583295	Norisoboldine 去甲异波尔定	C18H19NO4	313.348	HMDB0033357	NA	Alkaloids and derivatives	Aporphines	null	Alkaloids and derivatives	NA
485-72-3	9279782	7902313	9111916	Formononetin 刺芒柄花素	C16H12O4	268.264	HMDB0005808	C00858	Phenylpropanoids and polyketides	Isoflavonoids	O-methylated isoflavonoids	Flavonoids	NA
657-24-9	9104064	9137894	9865308	Metformin	C4H11N5	129.164	NA	C07151	Organic nitrogen compounds	Organonitrogen compounds	Guanidines	Guanidines	map04152 AMPK signaling pathway; map04211 Longevity regulating pathway; map04976 Bile secretion
100-42-5	8843823	7579583	8120820	Styrene	C8H8	104.149	HMDB0034240	C07083	Benzenoids	Benzene and substituted derivatives	Styrenes	Benzene and derivatives	NA
84104-71-2	8705471	9046675	8458173	Wilforlide A 雷公藤内酯甲	C30H46O3	454.684	NA	NA	NA	NA	NA	NA	NA
97938-30-2	8689518	8048279	7830044	Kushenol F 苦参醇F	C25H28O6	424.486	NA	NA	NA	NA	NA	NA	NA

6681-15-8	8573917	10735584	8511164	Jatrorrhizine hydrochloride 盐酸药根碱	C20H20ClNO4	374.838	NA	NA	NA	NA	NA	NA	NA
67-47-0	8530547	6530211	9025377	5-Hydroxymethyl droxymethyl-furfural 5-羟甲基糠醛	C6H6O3	126.11	HMDB0034355	C11101	Organic oxygen compounds	Organooxygen compounds	Carbonyl compounds	Aldehydes	NA
78415-72-2	7929304	8859231	9971677	Milrinone 米力农	C12H9N3O	211.219	HMDB0014380	C07224	Organohetero-cyclic compounds	Pyridines and derivatives	Bipyridines and oli-gopyridines	Pyridine and derivatives	NA
6873-13-8	7863698	6362080	7347694	Phellodendrine 黄柏碱	C20H24NO4	377.862	NA	C17046	NA	NA	NA	NA	NULL
1405-86-3	7755390	8221421	7629663	Glycyrrhizic acid 甘草酸	C42H62O16	822.932	HMDB0029843	NA	Lipids and lipid-like molecules	Prenol lipids	Terpene glycosides	Terpenoids	NA
486-63-5	7733165	8419731	7101519	4''-HYDROXY-7-METHOXY-ISOFLAVONE	C16H12O4	268.264	HMDB0033994	NA	Phenylpro-panoids and polyketides	Isoflavonoids	O-methylated isoflavonoids	Flavonoids	NA
33237-37-5	7681952	6665356	5588035	Rutaevin 吴茱萸苦素	C26H30O9	486.511	NA	C08779	NA	NA	NA	Terpenoids	NULL
23666-13-9	7618522	7797882	7625746	Vicenin II 维采宁II	C27H30O15	594.518	HMDB0030708	C10195	Phenylpro-panoids and polyketides	Flavonoids	Flavonoid glycosides	Flavonoids	NA
62-57-7	7582358	5942838	6496105	2-Aminoisobu-tyric acid	C4H9NO2	103.12	HMDB0001906	C03665	Organic acids and derivatives	Carboxylic acids and derivatives	Amino acids, peptides, and analogues	Amino acids, peptides, and analogues	NA
20362-31-6	7533316	7404324	11919471	Arctiin 牛蒡苷	C27H34O11	534.552	NA	C16915	NA	NA	NA	Lignans	NULL
476-66-4	7505578	6931762	7341904	Ellagic acid 鞣花酸	C14H6O8	302.193	HMDB0002899	C10788	Phenylpro-panoids and polyketides	Tannins	Hydrolyzable tannins	Tannins	NA
823214-06-8	7501916	7399727	11920821	Styraxlignolide F	C27H34O11	534.552	NA	NA	NA	NA	NA	NA	NA
20575-57-9	7148698	6893117	6225489	Calycosin 毛蕊异黄酮	C16H12O5	284.263	NA	C01562	NA	NA	NA	Flavonoids	map00943 Isoflavonoid biosynthesis; map01110 Biosynthesis of secondary metabolites
491-70-3	7095600	6574516	7198046	Luteolin 木犀草素	C15H10O6	286.236	HMDB0005800	C01514	Phenylpro-panoids and polyketides	Flavonoids	Flavones	Flavonoids	NA
10097-84-4	7033159	7261884	5530339	Rotundine 罗通定	C21H25NO4	355.427	NA	NA	NA	NA	NA	NA	NA
6024-85-7	7032315	7289525	5503067	Tetrahy-dropalmatine HCl 延胡索乙素	C21H26ClNO4	391.888	NA	NA	NA	NA	NA	NA	NA
3520-14-7	7000800	7270428	5489747	D-Tetrahy-dropalmatine 右旋四氢巴马汀	C21H25NO4	355.427	NA	NA	NA	NA	NA	NA	NA
2934-97-6	6967991	7236012	5476700	Tetrahy-dropalmatine 罗通定	C21H25NO4	355.427	NA	NA	NA	NA	NA	NA	NA
6882-68-4	6894879	8533769	7156730	Sophoridine 槐定碱	C15H24N2O	248.364	NA	NA	NA	NA	NA	NA	NA
520-27-4	6803933	6695385	5864932	Diosmin 地奥司明	C28H32O15	608.545	HMDB0029548	C10039	Phenylpro-panoids and polyketides	Flavonoids	Flavonoid glycosides	Flavonoids	NA
556-50-3	6568227	7016103	6004433	Glycylglycine	C4H8N2O3	132.118	HMDB0011733	C02037	Organic acids and derivatives	Carboxylic acids and derivatives	Amino acids, peptides, and analogues	Amino acids, peptides, and analogues	NA
4431-01-0	6505934	6266306	7279875	Ligustilide 藁本内酯	C12H14O2	190.238	HMDB0034277	C16987	Organohetero-cyclic compounds	Isobenzofurans	null	Furanones	NA
500-62-9	6456538	6961656	6961199	Yangonin	C15H14O4	258.269	HMDB0034144	C09980	Phenylpro-panoids and polyketides	Kavalactones	null	Polyke-tides[PK]	NA
13241-28-6	6415868	6509354	5959457	Chrysophanol 8-O-β-D-glucoside 大黄酚-8-O-β-D-葡萄糖苷	C21H20O9	416.378	HMDB0039098	C10316	Benzenoids	Anthracenes	Anthraqui-nones	Quinone	NA
80681-44-3	6352213	6170236	6184519	Sec-O-Gluco-sylhamaudol 亥茅酚苷	C21H26O10	438.425	NA	NA	NA	NA	NA	NA	NA
82475-03-4	6296938	5395720	6069356	Baicalin methyl ester 黄芩苷甲酯	C22H20O11	0	NA	NA	NA	NA	NA	NA	NA

1268798	6087085	6783886	6257440	Cynaroside	C21H20O11	448.377	HMDB0035588	C03951	Phenylpropanoids and polyketides	Flavonoids	Flavonoid glycosides	Flavonoids	NA
80286-58-4	6068090	9421935	3830808	artemisic acid	C15H22O2	234.334	NA	C20309	NA	NA	NA	NA	NULL
51-84-3	5991181	5916222	6880902	Acetylcholine	C7H16NO2+	146.207	HMDB0000895	C01996	Organic nitrogen compounds	Organonitrogen compounds	Quaternary ammonium salts	Quaternary ammonium salts	NA
14371-10-9	5850084	5726177	5195783	trans-Cinnamaldehyde	C9H8O	132.159	NA	NA	NA	NA	NA	NA	NA
737-52-0	5842412	5262515	4762892	Oxypeucedanin 氧化前胡素	C16H14O5	286.279	NA	NA	NA	NA	NA	NA	NA
490-83-5	5774354	5746543	6219991	Dehydroascorbic acid	C6H6O6	174.108	HMDB0001264	C05422	Organoheterocyclic compounds	Lactones	Gamma butyrolactones	Gamma butyrolactones	NA
437-64-9	5719712	5214530	5944065	Genkwanin 芫花素	C16H12O5	284.263	NA	C10046	NA	NA	NA	Flavonoids	NULL
478-01-3	5591127	5549432	6429071	Nobiletin 川陈皮素	C21H22O8	402.395	HMDB0029540	C10112	Phenylpropanoids and polyketides	Flavonoids	O-methylated flavonoids	Flavonoids	NA
73-24-5	5557007	5728171	5755573	Adenine 腺嘌呤	C5H5N5	135.127	HMDB0000034	C00147	Organoheterocyclic compounds	Imidazopyrimidines	Purines and purine derivatives	Purines and derivatives	NA
14941-08-3	5422541	6068144	4209371	Poncirin 枸橘苷	C28H34O14	594.561	HMDB0037487	C09830	Phenylpropanoids and polyketides	Flavonoids	Flavonoid glycosides	Flavonoids	NA
22884-10-2	5394038	6489381	5819315	1H-imidazole-1-acetic acid	C5H6N2O2	126.113	HMDB0029736	NA	Organic acids and derivatives	Carboxylic acids and derivatives	Amino acids, peptides, and analogues	Amino acids, peptides, and analogues	NA
15345-89-8	5321321	5151323	4198656	Demethoxyyangonin 去甲氧基醉椒素	C14H12O3	228.243	HMDB0034270	NA	Phenylpropanoids and polyketides	Kavalactones	null	Polyketides[PK]	NA
645-65-8	5213921	4227149	5781219	Imidazole-4-acetic acid	C5H6N2O2	126.113	HMDB0002024	C02835	Organoheterocyclic compounds	Azoles	Imidazoles	Imidazoles	NA
84676-89-1	5174215	4122370	4315529	Astragaloside II 黄芪皂苷II	C43H70O15	827.01	NA	C17798	NA	NA	NA	Terpenoids	NULL
512-69-6	5173621	4636768	3844869	Raffinose 棉籽糖	C18H32O16	504.437	HMDB0003213	C00492	Organic oxygen compounds	Organooxygen compounds	Carbohydrates and carbohydrate conjugates	Carbohydrates	NA
623-15-4	5058920	5397522	5136023	4-(2-Furyl)-3-buten-2-one	C8H8O2	136.148	HMDB0033128	NA	Organoheterocyclic compounds	Heteroaromatic compounds	null	Heteroaromatic compounds	NA
104-98-3	5006251	5907494	5424908	Urocanic acid	C6H6N2O2	138.124	NA	C00785	Organoheterocyclic compounds	Azoles	Imidazoles	Imidazoles	map00340 Histidine metabolism; map01100 Metabolic pathways
520-1+137:9362-7	4912267	4291675	4751004	Pectolinarigenin 柳穿鱼黄素	C17H14O6	314.289	NA	C17784	Phenylpropanoids and polyketides	Flavonoids	O-methylated flavonoids	Flavonoids	NULL
58-55-9	4871539	5196847	4859384	Theophylline	C7H8N4O2	180.164	HMDB0001889	C07130	Organoheterocyclic compounds	Imidazopyrimidines	Purines and purine derivatives	Purines and derivatives	NA
14259-47-3	4869606	5017573	4888166	Didymin 香蜂草苷	C28H34O14	594.561	HMDB0029482	NA	Phenylpropanoids and polyketides	Flavonoids	Flavonoid glycosides	Flavonoids	NA
6468-55-9	4863956	4286665	4524526	Demethylwedelolactone 去甲蟛蜞菊内酯	C15H8O7	300.22	NA	NA	NA	NA	NA	NA	NA
6894-43-5	4809052	4201936	3989337	Kahweol 咖啡豆醇	C20H26O3	314.419	HMDB0035602	NA	Lipids and lipid-like molecules	Prenol lipids	Monoterpenoids	Naphthofurans	NA
18059-10-4	4798846	4864668	5080770	Peiminine 贝母乙素	C27H43NO3	429.635	NA	NA	NA	NA	NA	NA	NA
137-08-6	4745848	13380664	2151093	Calcium pantothenate 泛酸钙	C9H16NO5 . 1/2 Ca	238.27	NA	C12276	NA	NA	NA	NA	NULL
608-66-2	4662196	5977445	6621913	Galactitol	C6H14O6	182.172	HMDB0000107	C01697	Organic oxygen compounds	Organooxygen compounds	Carbohydrates and carbohydrate conjugates	Carbohydrates	NA
552-54-5	4641076	4400673	4627035	Di-O-methylquercetin	C17H14O7	330.289	NA	NA	NA	NA	NA	NA	NA
106-60-5	4625791	4643732	4517353	5-Aminolevulinic acid	C5H9NO3	131.13	HMDB0001149	C00430	Organic acids and derivatives	Carboxylic acids and derivatives	Amino acids, peptides, and analogues	Amino acids, peptides, and analogues	NA
23496-41-5	4600588	4739530	5038409	Peimine	C27H45NO3	431.651	NA	C10830	NA	NA	NA	Alkaloids	NULL

				贝母甲素									
481-53-8	4525137	4003168	4523615	Tangeretin 桔皮素	C20H20O7	372.369	HMDB0030539	C10190	Phenylpropanoids and polyketides	Flavonoids	O-methylated flavonoids	Flavonoids	NA
327-57-1	4524693	3884657	3947080	L-Norleucine	C6H13NO2	131.173	HMDB0001645	C01933	Organic acids and derivatives	Carboxylic acids and derivatives	Amino acids, peptides, and analogues	Amino acids, peptides, and analogues	NA
99-96-7	4447854	4012933	4190472	4-Hydroxybenzoic acid 对羟基苯甲酸	C7H6O3	138.121	HMDB0000500	C00156	Benzenoids	Benzene and substituted derivatives	Benzoic acids and derivatives	Benzene and derivatives	NA
3943-97-3	4442669	3886066	3591764	Methyl 4-hydroxycinnamate 4-羟基肉桂酸甲酯	C10H10O3	178.185	NA	NA	NA	NA	NA	NA	NA
51-67-2	4430795	4539114	5232378	Tyramine	C8H11NO	137.179	HMDB0000306	C00483	Benzenoids	Benzene and substituted derivatives	Phenethylamines	Amines and derivatives	NA
486-66-8	4322876	4533902	5246116	Daidzein 大豆苷元	C15H10O4	254.238	HMDB0003312	C10208	Phenylpropanoids and polyketides	Isoflavonoids	Isoflav-2-enes	Flavonoids	NA
32507-66-7	4256364	5924696	6591376	Isorhapontigenin 异丹叶大黄素	C15H14O4	258.269	NA	NA	NA	NA	NA	NA	NA
38953-85-4	4107406	8649450	5196898	Isovitexin/Saponaretin 异牡荆黄素	C21H20O10	432.378	NA	C01714	NA	NA	NA	Flavonoids	map00944 Flavone and flavonol biosynthesis
27740-01-8	4076180	4418142	3827188	Scutellarin 野黄芩苷	C21H18O12	462.36	NA	NA	NA	NA	NA	NA	NA
73-32-5	4071868	4277741	5181888	L-Isoleucine	C6H13NO2	131.173	HMDB0000172	C00407	Organic acids and derivatives	Carboxylic acids and derivatives	Amino acids, peptides, and analogues	Amino acids	NA
119-84-6	4064935	3426909	4289221	Dihydrocoumarin	C9H8O2	148.159	HMDB0036626	C02274	Phenylpropanoids and polyketides	3,4-dihydrocoumarins	null	Coumarins and derivatives	NA
65-46-3	3984698	4930937	3940061	Cytidine 胞苷	C9H13N3O5	243.217	HMDB0000089	C00475	Nucleosides, nucleotides, and analogues	Pyrimidine nucleosides	null	Nucleic acids and analogues	NA
29700-22-9	3784472	3708215	3806128	Oxyresveratrol 氧化白藜芦醇	C14H12O4	244.243	NA	C10273	Phenylpropanoids and polyketides	Stilbenes	null	Polyketides[PK]	NULL
10083-24-6	3773032	3667689	3803358	Piceatannol 白皮杉醇	C14H12O4	244.243	HMDB0004215	C05901	Phenylpropanoids and polyketides	Stilbenes	null	Polyketides[PK]	NA
446-72-0	3766057	3738350	3458706	Genistein 染料木素	C15H10O5	270.237	HMDB0003217	C06563	Phenylpropanoids and polyketides	Isoflavonoids	Isoflav-2-enes	Flavonoids	NA
469-59-0	3691838	2362571	2630315	Jervine 蒜藜芦碱	C27H39NO3	425.603	NA	C10811	NA	NA	NA	Alkaloids	map01066 Biosynthesis of alkaloids derived from terpenoid and polyketide
25368-11-0	3675979	3278426	3567762	Asperulosidic acid 车叶草苷酸	C18H24O12	432.376	NA	NA	NA	NA	NA	NA	NA
13382-86-0	3647151	2843721	4802629	Manninotriose 甘露三糖	C18H32O16	504.437	NA	C05404	Organic oxygen compounds	Organooxygen compounds	Carbohydrates and carbohydrate conjugates	Carbohydrates	map00052 Galactose metabolism; map01100 Metabolic pathways
87-32-1	3576303	3422814	3291157	N-Acetyl-DL-tryptophan	C13H14N2O3	246.262	NA	NA	NA	NA	NA	NA	NA
29741-10-4	3503659	4508214	3283510	Luteolin 7-glucuronide 木犀草素-7-葡萄糖醛酸苷	C21H18O12	462.36	NA	NA	NA	NA	NA	NA	NA
92-61-5	3497529	5090911	5236709	Scopoletin 东莨菪内酯	C10H8O4	192.168	HMDB0034344	C01752	Phenylpropanoids and polyketides	Coumarins and derivatives	Hydroxycoumarins	Coumarins and derivatives	NA
585-88-6	3469774	3835257	3747751	Maltitol	C12H24O11	344.312	HMDB0002928	NA	Lipids and lipid-like molecules	Fatty Acyls	Fatty acyl glycosides	Fatty acyls[FA]	NA
7061-54-3	3462244	3683290	3970894	Phloridzin 根皮苷	C21H28O12	472.44	NA	NA	NA	NA	NA	NA	NA
60-27-5	3437945	3658925	3767526	Creatinine	C4H7N3O	113.118	HMDB0000562	C00791	Organic acids and derivatives	Carboxylic acids and derivatives	Amino acids, peptides, and analogues	Amino acids, peptides, and analogues	NA
123-99-9	3383484	2527874	2382149	Azelaic acid 壬二酸	C9H16O4	188.221	HMDB0000784	C08261	Lipids and lipid-like molecules	Fatty Acyls	Fatty acids and conjugates	Fatty acyls[FA]	NA

52617-37-5	3379545	3525094	3075549	Ponicidin/Rubescensin B 冬凌草乙素	C20H26O6	362.417	NA	NA	NA	NA	NA	NA	NA
64820-99-1	3367052	3602200	3157564	Vitexin rhamnoside 牡荆素鼠李糖苷	C27H30O14	578.519	NA	C12628	NA	NA	NA	Flavonoids	map00944 Flavone and flavonol biosynthesis
40246-10-4	3343206	3272881	3591003	Glycitin 黄豆黄苷	C22H22O10	446.404	NA	C16195	NA	NA	NA	Flavonoids	map00943 Isoflavonoid biosynthesis
24211-30-1	3316105	3192769	3377510	Farrerol 杜鹃素	C17H16O5	300.306	NA	C09734	NA	NA	NA	Flavonoids	NULL
1180-71-8	3237372	3220275	2799952	Limonin 柠檬苦素	C26H30O8	470.512	HMDB0035921	C03514	Lipids and lipid-like molecules	Prenol lipids	Triterpenoids	Terpenoids	NA
137592-12-2	3207919	2814928	3377540	Taxifolin 7-rhamnoside 二氢田基黄苷	C21H22O11	450.393	NA	NA	NA	NA	NA	NA	NA
537-98-4	3176368	2989098	2761059	(E)-Ferulic acid	C10H10O4	194.184	HMDB0000954	C01494	Phenylpropanoids and polyketides	Cinnamic acids and derivatives	Hydroxycinnamic acids and derivatives	Polyketides[PK]	NA
473-08-5	3175816	3513409	4429481	α-Cyperone α-香附酮	C15H22O	218.335	HMDB0037061	C17090	Lipids and lipid-like molecules	Prenol lipids	Sesquiterpenoids	Terpenoids	NA
20554-84-1	3046190	2670697	2967882	Parthenolide 小白菊内酯	C15H20O3	248.318	NA	C07609	NA	NA	NA	Terpenoids	NULL
602-41-5	3015220	2792356	2354613	Thiocolchicoside 硫秋水仙苷	C27H33NO10S	563.617	NA	NA	NA	NA	NA	NA	NA
564-20-5	3004413	7520006	5835174	Clareolide 香紫苏内酯	C16H26O2	250.376	HMDB0035293	NA	Organoheterocyclic compounds	Naphthofurans	null	Naphthofurans	NA
71-30-7	2947207	3050159	3160804	Cytosine 胞嘧啶	C4H5N3O	111.102	HMDB0000630	C00380	Organoheterocyclic compounds	Diazines	Pyrimidines and pyrimidine derivatives	Pyrimidines and pyrimidine derivatives	NA
60-81-1	2933532	2850400	3142068	Phlorizin	C21H24O10	436.409	HMDB0036634	C01604	Phenylpropanoids and polyketides	Flavonoids	Flavonoid glycosides	Flavonoids	NA
196618-13-0	2903904	3644988	3111328	Oseltamivir	C16H28N2O4	312.405	NA	C08092	NA	NA	NA	Amino acids, peptides, and analogues	NULL
552-59-0	2869925	2836128	2312641	Prunetin 樱黄素	C16H12O5	284.263	HMDB0034127	C10521	Phenylpropanoids and polyketides	Isoflavonoids	O-methylated isoflavonoids	Flavonoids	NA
66-22-8	2819903	2905586	2737019	Uracil	C4H4N2O2	112.087	HMDB0000300	C00106	Organoheterocyclic compounds	Diazines	Pyrimidines and pyrimidine derivatives	Pyrimidines and pyrimidine derivatives	NA
87-79-6	2759358	3419340	3298905	L-Sorbose	C6H12O6	180.156	NA	C00247	NA	NA	NA	Carbohydrates	map00051 Fructose and mannose metabolism; map01100 Metabolic pathways
84954-92-7	2755609	3066346	3243027	Rosavin 络塞维	C20H28O10	428.43	NA	NA	NA	NA	NA	NA	NA
134859-96-4	2733085	2989225	2361549	6"-O-Acetylglycitin 6"-O-乙酰黄豆黄苷	C24H24O11	488.441	HMDB0039489	NA	Phenylpropanoids and polyketides	Isoflavonoids	Isoflavonoid O-glycosides	Flavonoids	NA
487-41-2	2732416	2622244	3620851	Forsythin 连翘苷	C27H34O11	534.552	NA	C17048	NA	NA	NA	Lignans	NULL
65-86-1	2727681	2627138	2560787	Orotic acid	C5H4N2O4	156.096	HMDB0000226	C00295	Organoheterocyclic compounds	Diazines	Pyrimidines and pyrimidine derivatives	Pyrimidines and pyrimidine derivatives	NA
98-86-2	2699983	2715985	2200497	Acetophenone 乙酰苯	C8H8O	120.148	HMDB0033910	C07113	Organic oxygen compounds	Organooxygen compounds	Carbonyl compounds	Carbonyl compounds	NA
19431-84-6	2677742	2666697	3379958	Curcumenol 莪术烯醇	C15H22O2	234.33	HMDB0033960	NA	Lipids and lipid-like molecules	Prenol lipids	Sesquiterpenoids	Terpenoids	NA
480-44-4	2633138	2833734	2489637	Acacetin 金合欢素	C16H12O5	284.263	NA	C01470	Phenylpropanoids and polyketides	Flavonoids	O-methylated flavonoids	Flavonoids	map00944 Flavone and flavonol biosynthesis
1946-82-3	2616783	2299883	2665766	N-α-Acetyl-L-lysine	C8H16N2O3	188.224	HMDB0000446	C12989	Organic acids and derivatives	Carboxylic acids and derivatives	Amino acids, peptides, and analogues	Amino acids, peptides, and analogues	NA
128397-41-1	2597713	1924402	2098643	Hydroprotopine 氢化原阿片碱	C20H20NO5+	354.377	NA	NA	NA	NA	NA	NA	NA
484-29-7	2576599	2701273	2653417	Dictamnine	C12H9NO2	199.205	NA	C10660	NA	NA	NA	Alkaloids	map01063 Biosynthesis of

				白鲜碱									alkaloids derived from shikimate pathway
2490-97-3	2534823	2375158	1861225	N-α-Acetyl-L-glutamine	C7H12N2O4	188.181	HMDB0006029	NA	Organic acids and derivatives	Carboxylic acids and derivatives	Amino acids, peptides, and analogues	Amino acids, peptides, and analogues	NA
21422-04-8	2521607	2570993	2183005	7-Demethylsuberosin 7-去甲基软木花椒素	C14H14O3	230.259	NA	C18083	NA	NA	NA	NA	map01061 Biosynthesis of phenylpropanoids
17575-22-3	2503871	1141046	783432	Lanatoside C 毛花苷C	C49H76O20	985.116	NA	C13470	NA	NA	NA	NA	NULL
551-08-6	2493326	2174216	2466823	3-Butylidenephthalide 丁烯基苯酞	C12H12O2	188.223	HMDB0032061	C16924	Organoheterocyclic compounds	Isocoumarans	Isobenzofuranones	Isobenzofuranones	NA
520-34-3	2480931	2453967	2571657	Diosmetin 香叶木素	C16H12O6	300.263	HMDB0029676	C10038	Phenylpropanoids and polyketides	Flavonoids	O-methylated flavonoids	Flavonoids	NA
37921-38-3	2467091	2221523	2253779	Cimifugin 升麻素	C16H18O6	306.31	NA	C09000	NA	NA	NA	Polyketides[PK]	NULL
104594-70-9	2453451	2370269	2225871	Phenethyl caffeate 咖啡酸苯乙酯	C17H16O4	284.306	NA	C10484	NA	NA	NA	NA	NULL
487-06-9	2446739	2226657	1442483	Citropten 白柠檬素	C11H10O4	206.195	HMDB0032952	NA	Phenylpropanoids and polyketides	Coumarins and derivatives	null	Coumarins and derivatives	NA
516-05-2	2434600	2496935	2089086	2-Methylmalonic acid	C4H6O4	118.088	HMDB0000202	C02170	Organic acids and derivatives	Carboxylic acids and derivatives	Dicarboxylic acids and derivatives	Organic acids	NA
52-53-9	2431025	2651919	3062215	Verapamil	C27H38N2O4	454.602	HMDB0001850	C07188	Benzenoids	Benzene and substituted derivatives	Phenylbutylamines	Amines and derivatives	NA
102067-92-5	2430903	2623460	5221818	Epi-theaflagallin 3-O-gallate	C27H20O13	552.4399	HMDB0033288	NA	Benzenoids	Benzene and substituted derivatives	Benzoic acids and derivatives	Benzene and derivatives	NA
4191-73-5	2413701	2627342	2214168	Isopropyl 4-Hydroxybenzoate 尼泊金异丙酯	C10H12O3	180.201	NA	C20343	NA	NA	NA	NA	NULL
18422-05-4	2370235	1860798	2012177	Adenosine 5-monophosphate	C10H16N5O8P	365.237	NA	NA	NA	NA	NA	NA	NA
526-99-8	2362896	2492383	2641897	Mucic acid	C6H10O8	210.139	HMDB0000639	C00879	Organic oxygen compounds	Organooxygen compounds	Carbohydrates and carbohydrate conjugates	Carbohydrates	NA
4046-02-0	2349888	2006401	2381351	Ethyl ferulate 阿魏酸乙酯	C12H14O4	222.24	NA	NA	NA	NA	NA	NA	NA
118-71-8	2264667	2043473	2403224	3-Hydroxy-2-methyl-4-pyrone	C6H6O3	126.11	HMDB0030776	C11918	Organoheterocyclic compounds	Pyrans	Pyranones and derivatives	Pyranones and derivatives	NA
298-46-4	2208853	2047399	1984186	Carbamazepine	C15H12N2O	236.269	HMDB0014704	C06868	Organoheterocyclic compounds	Benzazepines	Dibenzazepines	Dibenzazepines	NA
58-61-7	2194023	2375128	2118951	Adenosine 腺苷	C10H13N5O4	267.241	HMDB0000050	C00212	Nucleosides, nucleotides, and analogues	Purine nucleosides	null	Purines and derivatives	NA
520-28-5	2177837	2148340	2017278	Tectochrysin 杨芽黄素	C16H12O4	268.264	NA	C11621	NA	NA	NA	Flavonoids	NULL
500-65-2	2150695	2155977	2547095	Rhapontigenin 丹叶大黄素	C15H14O4	258.269	HMDB0031842	NA	Phenylpropanoids and polyketides	Stilbenes	null	Polyketides[PK]	NA
32981-86-5	2143512	2080077	1585351	10-Deacetylbaccatin III 10-脱乙酰巴卡亭III	C29H36O10	544.59	NA	C11700	NA	NA	NA	Terpenoids	map00904 Diterpenoid biosynthesis; map01100 Metabolic pathways; map01110 Biosynthesis of secondary metabolites
6151-40-2	2142833	1948564	2420439	Quinidine HCl 奎尼丁盐酸盐	C20H27ClN2O3	378.893	NA	NA	NA	NA	NA	NA	NA
153355-70-5	2117405	1998294	1579747	Ginkgolide K 银杏内酯k	C20H22O9	406.383	NA	NA	NA	NA	NA	NA	NA
574-84-5	2082242	2627806	2442820	Fraxetin 秦皮素	C10H8O5	208.167	NA	C09265	NA	NA	NA	Coumarins	NULL
1447-88-7	2063284	1991399	2301307	Hispidulin 高车前素	C16H12O6	300.263	NA	C10058	Phenylpropanoids and polyketides	Flavonoids	O-methylated flavonoids	Flavonoids	NULL
5610-40-2	2060428	2388188	2195830	Securinine	C13H15NO2	217.264	NA	C10614	NA	NA	NA	NA	NULL

				一叶萩碱									
548-77-6	2056003	1988285	2247807	Tectorigenin 射干苷元	C16H12O6	300.263	HMDB0042024	C10534	Phenylpropanoids and polyketides	Isoflavonoids	Isoflav-2-enes	Flavonoids	NA
480-40-0	2055401	1839677	1894241	Chrysin 白杨素	C15H10O4	254.238	HMDB0036619	C10028	Phenylpropanoids and polyketides	Flavonoids	Flavones	Flavonoids	NA
2555-28-4	2051231	1760816	2367402	7-Methoxy-4-methylcoumarin 7-甲氧基-4-甲基香豆素	C11H10O3	190.195	NA	NA	NA	NA	NA	NA	NA
144-11-6	2045273	1968076	2106687	Benzhexol	C20H31NO	301.466	HMDB0014520	C07171	Organic nitrogen compounds	Organonitrogen compounds	Amines	Amines	NA
41060-15-5	2016043	1851431	1851456	Neobavaisoflavone 新补骨脂异黄酮	C20H18O4	322.354	NA	NA	NA	NA	NA	NA	NA
54010-71-8	1997440	1699237	1912078	D-Glucose 6-phosphate	C6H12NaO9P	282.118	NA	NA	NA	NA	NA	NA	NA
17680-84-1	1980741	2398588	1645555	Homo-plantaginin 高车前苷	C22H22O11	462.404	NA	NA	NA	NA	NA	NA	NA
88-99-3	1952445	1675614	1689235	Phthalic acid	C8H6O4	166.131	HMDB0002107	C01606	Benzenoids	Benzene and substituted derivatives	Benzoic acids and derivatives	Benzene and derivatives	NA
55-22-1	1908747	2114209	1836952	Isonicotinic acid	C6H5NO2	123.109	HMDB0060665	C07446	Organoheterocyclic compounds	Pyridines and derivatives	Pyridinecarboxylic acids and derivatives	Pyridine and derivatives	NA
60-18-4	1901335	2112206	2091321	L-Tyrosine L-酪氨酸	C9H11NO3	181.189	HMDB0000158	C00082	Organic acids and derivatives	Carboxylic acids and derivatives	Amino acids, peptides, and analogues	Amino acids	NA
531-59-9	1900396	1627880	1620434	7-Methoxycoumarin 甲氧基香豆素	C10H8O3	176.169	HMDB0029758	C09268	Phenylpropanoids and polyketides	Coumarins and derivatives	null	Coumarins and derivatives	NA
2450-53-5	1877066	1820805	2006978	3,5-Dicaffeoylquinic acid 异绿原酸A	C25H24O12	516.451	HMDB0030706	NA	Organic oxygen compounds	Organooxygen compounds	Alcohols and polyols	Alcohols	NA
10516-71-9	1869752	1699836	1905058	3-(3-Methoxyphe-phe-nyl)propionic acid	C10H12O3	180.201	HMDB0011751	NA	Phenylpropanoids and polyketides	Phenylpropanoic acids	null	Phenylpropanoic acids	NA
476-32-4	1861319	1444718	1365990	Chelidonine 白屈菜碱	C20H19NO5	353.369	NA	C12242	NA	NA	NA	Alkaloids	NULL
10401-33-9	1811634	1634668	1654921	Glabrolide 光甘草内酯	C30H44O4	468.668	HMDB0034515	NA	Lipids and lipid-like molecules	Prenol lipids	Triterpenoids	Terpenoids	NA
482-38-2	1805774	2029500	1792022	Kaempferitrin 山奈苷	C27H30O14	578.519	HMDB0037438	C16981	Phenylpropanoids and polyketides	Flavonoids	Flavonoid glycosides	Flavonoids	NA
94367-42-7	1801608	1809912	1937939	Methylnissolin-3-O-glucoside 美迪紫檀苷	C23H26O10	462.447	NA	NA	NA	NA	NA	NA	NA
110-16-7	1797090	2319467	2078225	Maleic acid 马来酸	C4H4O4	116.072	HMDB0000176	C01384	Organic acids and derivatives	Carboxylic acids and derivatives	Dicarboxylic acids and derivatives	Organic acids	NA
491-69-0	1792809	1162913	1408062	Isosakuranin 异樱花苷	C22H24O10	448.42	HMDB0029481	NA	Phenylpropanoids and polyketides	Flavonoids	Flavonoid glycosides	Flavonoids	NA
14534-61-3	1774664	1367237	909295	Isochlorogenic acid B 异绿原酸B	C25H24O12	516.451	HMDB0030705	C10468	Organic oxygen compounds	Organooxygen compounds	Alcohols and polyols	Alcohols	NA
19950-55-1	1736531	1675124	1724348	Veraguensin 蔚瑞昆森	C22H28O5	372.455	NA	C10892	NA	NA	NA	Lignans	NULL
23627-87-4	1720545	1518367	1898912	Kaempferol 3-O-galactoside (Trifolin)	C21H20O11	448.377	HMDB0030864	C12626	Phenylpropanoids and polyketides	Flavonoids	Flavonoid glycosides	Flavonoids	NA
140147-77-9	1715711	1520946	1372783	Epimedin A1 朝藿定A1	C39H50O20	838.802	NA	NA	NA	NA	NA	NA	NA
31524-62-6	1713143	1513204	1686423	Isobavachin 异补骨脂二氢黄酮	C20H20O4	324.37	NA	NA	NA	NA	NA	NA	NA
491-71-4	1694079	1739147	1673459	Chrysoeriol	C16H12O6	300.263	HMDB0030667	C04293	Phenylpropanoids and polyketides	Flavonoids	O-methylated flavonoids	Flavonoids	NA
298-81-7	1693912	1507847	1950739	8-Methoxypsoralen 花椒毒素	C12H8O4	216.189	HMDB0014693	C01864	Phenylpropanoids and polyketides	Coumarins and derivatives	Furanocoumarins	Coumarins and derivatives	NA
110623-72-8	1689140	1516994	1384286	Epimedin A	C39H50O20	838.8	NA	NA	NA	NA	NA	NA	NA

				朝藿定A									
72-48-0	1673383	1370535	1473952	1,2-Dihydroxy anthraquinone 茜素	C14H8O4	240.211	NA	C01474	NA	NA	NA	Quinone	NULL
77026-92-7	1658942	1718364	1679412	trans-Jasmonic Acid	C12H18O3	196.243	NA	NA	NA	NA	NA	NA	NA
66-72-8	1656284	1504797	1570202	Pyridoxal	C8H9NO3	167.162	HMDB0001545	C00250	Organohetero-cyclic compounds	Pyridines and derivatives	Pyridine carboxalde-hydes	Pyridine and derivatives	NA
7770-78-7	1653989	1405068	2256503	Arctigenin 牛蒡子苷元	C21H24O6	372.412	HMDB0030087	C10545	Lignans, neolignans and related compounds	Furanoid lignans	Tetrahydrofu-ran lignans	Lignans and related compounds	NA
487-39-8	1649137	1477818	2287989	Phillygenin 连翘脂素	C21H24O6	372.412	NA	NA	NA	NA	NA	NA	NA
905-99-7	1646440	1473188	1625905	Cryptochloro-genic acid 隐绿原酸	C16H18O9	354.309	HMDB0030653	NA	Organic oxygen compounds	Organooxygen compounds	Alcohols and polyols	Alcohols	NA
2244-16-8	1637159	1579698	1304209	(+)-(S)-Carvone	C10H14O	150.218	HMDB0004487	C11383	Lipids and lipid-like molecules	Prenol lipids	Monoterpe-noids	Terpenoids	NA
24512-62-7	1635603	1543196	1681264	Gardenoside 羟异栀子苷	C17H24O11	404.366	NA	C09779	NA	NA	NA	Terpenoids	NULL
55033-90-4	1614066	1254868	1582482	Isorhamnetin-3-O-nehesperidine 异鼠李素-3-O-新橙皮苷	C28H32O16	624.544	HMDB0037745	NA	Phenylpro-panoids and polyketides	Flavonoids	Flavonoid glycosides	Flavonoids	NA
604-80-8	1607079	1356211	1378380	Narcissoside 水仙苷	C28H32O16	624.544	NA	NA	NA	NA	NA	NA	NA
480-10-4	1606051	1609365	1516609	Astragalin 紫云英苷	C21H20O11	448.377	HMDB0037429	C12249	Phenylpro-panoids and polyketides	Flavonoids	Flavonoid glycosides	Flavonoids	NA
16290-07-6	1606051	1609365	1516609	Kaempferol-7-O-β-D-glucopyra-noside 山奈酚-7-O-β-D-葡萄糖苷	C21H20O11	448.377	NA	NA	NA	NA	NA	NA	NA
98-98-6	1600363	1550979	1719165	2-Picolinic acid	C6H5NO2	123.109	HMDB0002243	C10164	Organohetero-cyclic compounds	Pyridines and derivatives	Pyridinecar-boxylic acids and derivatives	Pyridine and derivatives	NA
37308-75-1	1599331	1582227	1460226	Flavokawain C 黄卡瓦胡椒素 C	C17H16O5	300.306	HMDB0030875	NA	Phenylpro-panoids and polyketides	Linear 1,3-diarylpro-panoids	Chalcones and dihydrochal-cones	Polyke-tides[PK]	NA
67416-61-9	1572021	1469836	1508668	Acetyl-11-keto-β-boswellic acid 乙酰基-11-酮基-β-乳香酸	C32H48O5	512.721	HMDB0036672	NA	Lipids and lipid-like molecules	Prenol lipids	Triterpenoids	Terpenoids	NA
597-43-3	1560998	1298875	1388475	2,2-Dimethylsuc-cinic acid	C6H10O4	146.141	HMDB0002074	NA	Lipids and lipid-like molecules	Fatty Acyls	Fatty acids and conjugates	Fatty acyls[FA]	NA
327-97-9	1552472	1401151	1505326	Chlorogenic acid 绿原酸	C16H18O9	354.309	HMDB0003164	NA	Organic oxygen compounds	Organooxygen compounds	Alcohols and polyols	Alcohols	NA
623-30-3	1545051	1258456	1271636	Furylacrolein	C7H6O2	122.121	HMDB0032918	NA	Organohetero-cyclic compounds	Heteroaromatic compounds	null	Heteroaromatic compounds	NA
303-45-7	1539024	1518655	1464735	Gossypol 棉酚	C30H30O8	518.554	NA	C07667	Lipids and lipid-like molecules	Prenol lipids	Sesquiterpe-noids	Terpenoids	map00909 Sesquiterpe-noid and triterpenoid biosynthesis; map01062 Biosynthesis of terpenoids and steroids; map01110 Biosynthesis of secondary metabolites
3785-24-8	1522249	1391950	1997414	Nepodin 羊蹄素	C13H12O3	216.233	NA	C09954	NA	NA	NA	NA	NULL
491-74-7	1514697	1503263	1483782	Iridin 野鸢尾苷	C24H26O13	522.455	NA	C10465	NA	NA	NA	Flavonoids	NULL
522-12-3	1511732	1951940	856723	Quercitrin 槲皮甙	C21H20O11	448.377	HMDB0033751	C01750	Phenylpro-panoids and polyketides	Flavonoids	Flavonoid glycosides	Flavonoids	NA
23513-14-6	1494474	1434616	1369724	6-Gingerol 6-姜酚	C17H26O4	294.386	NA	C10462	NA	NA	NA	Polyke-tides[PK]	map00945 Stilbenoid, diarylheptanoid and gingerol biosynthesis; map01110 Biosynthesis of secondary

													metabolites
59-67-6	1486871	1268882	1368158	Nicotinic acid 烟酸	C6H5NO2	123.11	HMDB0001488	C00253	Organoheterocyclic compounds	Pyridines and derivatives	Pyridinecarboxylic acids and derivatives	Pyridine and derivatives	NA
19902-91-1	1472386	1590805	1617407	Dihydromethysticin 二氢麻醉椒苦素	C15H16O5	276.284	HMDB0030791	C09926	Phenylpropanoids and polyketides	Kavalactones	null	Polyketides[PK]	NA
520-33-2	1439202	1688684	1573212	Hesperetin 橙皮素	C16H14O6	302.279	HMDB0005782	C01709	Phenylpropanoids and polyketides	Flavonoids	O-methylated flavonoids	Flavonoids	NA
89-57-6	1425929	915053	1215634	5-Aminosalicylic acid	C7H7NO3	153.135	HMDB0014389	C07138	Benzenoids	Benzene and substituted derivatives	Benzoic acids and derivatives	Benzene and derivatives	NA
13133-07-8	1425347	1296173	1495953	Nystose 耐斯糖	C24H42O21	666.578	HMDB0039176	NA	Organic oxygen compounds	Organooxygen compounds	Carbohydrates and carbohydrate conjugates	Carbohydrates	NA
110-17-8	1394084	2570704	1801779	Fumaric acid 富马酸	C4H4O4	116.072	HMDB0000134	C00122	Organic acids and derivatives	Carboxylic acids and derivatives	Dicarboxylic acids and derivatives	Organic acids	NA
97-14-3	1387067	1442965	1390566	N-Acetyl-DL-serine	C5H9NO4	147.129	NA	NA	NA	NA	NA	NA	NA
97230-47-2	1381983	1176439	1357898	Picfeltarraenin IA 苦玄参苷IA	C41H62O13	762.923	NA	NA	NA	NA	NA	NA	NA
491-67-8	1376993	1195690	1403222	Baicalein 黄芩素	C15H10O5	270.237	NA	C10023	Phenylpropanoids and polyketides	Flavonoids	Flavones	Flavonoids	NULL
42206-94-0	1375100	1382889	1377904	Acetylresveratrol 乙酰白藜芦醇	C20H18O6	354.353	NA	NA	NA	NA	NA	NA	NA
1156-78-1	1372470	1231321	1219236	2'-Hydroxygenistein	C15H10O6	286.236	HMDB0034014	C12134	Phenylpropanoids and polyketides	Isoflavonoids	Isoflav-2-enes	Flavonoids	NA
1072-93-1	1364406	1365137	1665314	Epigoitrin 表告依春	C5H7NOS	129.18	NA	NA	NA	NA	NA	NA	NA
463-00-3	1344489	1697085	1463788	4-Guanidinobutyric acid	C5H11N3O2	145.16	HMDB0003464	NA	Organic acids and derivatives	Carboxylic acids and derivatives	Amino acids, peptides, and analogues	Amino acids, peptides, and analogues	NA
129025-96-3	1344246	1348013	1599149	Aurantio-obtusin β-D-glucoside 橙黄决明素-6-O-葡萄糖苷	C23H24O12	492.43	NA	C10303	NA	NA	NA	Quinone	NULL
1798-09-0	1338523	1218294	1820135	3-Methoxyphenylacetic acid	C9H10O3	166.174	HMDB0059969	NA	Benzenoids	Phenol ethers	Anisoles	Benzene and derivatives	NA
522-97-4	1338454	1303738	1116670	Tetrahydroberberine THB 四氢小檗碱	C20H21NO4	339.385	NA	NA	NA	NA	NA	NA	NA
480-43-3	1335511	1172268	1109623	Isosakuranetin 异樱花素	C16H14O5	286.279	NA	C05334	NA	NA	NA	Flavonoids	map00941 Flavonoid biosynthesis; map01110 Biosynthesis of secondary metabolites
56-86-0	1332646	1227512	950797	L-Glutamic acid L-谷氨酸	C5H9NO4	147.129	HMDB0000148	C00025	Organic acids and derivatives	Carboxylic acids and derivatives	Amino acids, peptides, and analogues	Amino acids	NA
6066-49-5	1330649	1347401	1533204	3-n-Butylphathlide 正丁基苯酞	C12H14O2	190.238	NA	C17854	Organoheterocyclic compounds	Benzofurans	Benzofuranones	Benzofuranones	NULL
149155-01-1	1325235	1064072	1190691	Cratoxylone 黄牛木酮	C24H28O7	428.475	NA	NA	NA	NA	NA	NA	NA
490-46-0	1318534	1730828	1263556	Epicatechin 表儿茶素	C15H14O6	290.268	HMDB0001871	C09727	Phenylpropanoids and polyketides	Flavonoids	Flavans	Flavonoids	NA
3549-23-3	1316837	1341766	1407685	Methyl p-tert-butylphenylacetate	C13H18O2	206.281	HMDB0036240	NA	Benzenoids	Benzene and substituted derivatives	Phenylpropanes	Benzene and derivatives	NA
56-25-7	1316401	1340747	1477031	Cantharidin 斑蝥素	C10H12O4	196.2	NA	C16778	NA	NA	NA	NA	NULL
13463-28-0	1309019	1705954	1844176	Eriocitrin 圣草次苷	C27H32O15	596.534	HMDB0005811	C09732	Benzenoids	Naphthalenes	Phenylnaphthalenes	Benzene and derivatives	NA
464-74-4	1308631	1263832	1019854	Arenobufagin 沙蟾蜍精	C24H32O6	416.507	NA	C20035	NA	NA	NA	Venoms	NULL
60008-02-8	1306888	1291042	1262796	Glabrone 光甘草酮	C20H16O5	336.338	HMDB0029533	NA	Phenylpropanoids and polyketides	Isoflavonoids	Pyranoisoflavonoids	Flavonoids	NA

97792-45-5	1305100	1189576	1392722	Lappaconitine hydrobromide 氢溴酸高乌甲素	$C_{32}H_{45}BrN_2O_8$	665.612	NA	NA	NA	NA	NA	NA	NA
20126-59-4	1304253	1434526	1031933	Diosmetin-7-O-β-D-glucopyranoside 香叶木素-7-O-β-D-葡萄糖苷	$C_{22}H_{22}O_{11}$	462.404	HMDB0037451	NA	Phenylpropanoids and polyketides	Flavonoids	Flavonoid glycosides	Flavonoids	NA
479-91-4	1303271	1133524	1386247	Casticin 蔓荆子黄素	$C_{19}H_{18}O_8$	374.341	HMDB0030660	NA	Phenylpropanoids and polyketides	Flavonoids	O-methylated flavonoids	Flavonoids	NA
603-56-5	1303271	1133524	1386247	Chrysosplenetin B 金腰乙素	$C_{19}H_{18}O_8$	374.341	NA	C10030	NA	NA	NA	Flavonoids	NULL
69651-80-5	1273889	1378480	1177648	Hesperetin 5-O-glucoside	$C_{22}H_{24}O_{11}$	464.419	HMDB0037535	NA	Phenylpropanoids and polyketides	Flavonoids	Flavonoid glycosides	Flavonoids	NA
50-67-9	1272886	1308221	1394395	Serotonin	$C_{10}H_{12}N_2O$	176.215	HMDB0000259	C00780	Organoheterocyclic compounds	Indoles and derivatives	Tryptamines and derivatives	Amines and derivatives	NA
104-14-3	1268496	1221371	1287117	Octopamine	$C_8H_{11}NO_2$	153.178	HMDB0004825	C04227	Benzenoids	Phenols	1-hydroxy-2-unsubstituted benzenoids	Phenols and derivatives	NA
17306-46-6	1260904	1033852	1576161	Rhoifolin 野漆树苷	$C_{27}H_{30}O_{14}$	578.519	HMDB0038848	C12627	Phenylpropanoids and polyketides	Flavonoids	Flavonoid glycosides	null	NA
487-03-6	1258521	1127478	1300465	Harmol 哈尔酚	$C_{12}H_{10}N_2O$	198.221	HMDB0034217	NA	Alkaloids and derivatives	Harmala alkaloids	null	Alkaloids and derivatives	NA
19408-84-5	1257975	1284880	1375754	Dihydrocapsaicin 二氢辣椒素	$C_{18}H_{29}NO_3$	307.428	HMDB0038457	C16952	Benzenoids	Phenols	Methoxyphenols	Phenols and derivatives	NA
76-22-2	1252322	1588881	1361536	Camphor 樟脑	$C_{10}H_{16}O$	152.233	NA	C18369	NA	NA	NA	Terpenoids	NULL
20183-47-5	1251779	1406859	1338376	Tenuifolin 细叶远志皂苷	$C_{36}H_{56}O_{12}$	680.823	NA	NA	NA	NA	NA	NA	NA
92-48-8	1248018	1198144	951718	6-Methylcoumarin	$C_{10}H_8O_2$	160.169	HMDB0032394	NA	Phenylpropanoids and polyketides	Coumarins and derivatives	null	Coumarins and derivatives	NA
149567	1246356	1087212	1227112	Ferulic acid methyl ester	$C_{11}H_{12}O_4$	208.21	NA	NA	NA	NA	NA	NA	NA
58-27-5	1239524	1744445	1841530	Menadione	$C_{11}H_8O_2$	172.18	HMDB0001892	C05377	Benzenoids	Naphthalenes	Naphthoquinones	Quinone	NA
63492-69-3	1236508	1395467	1681899	Lithosprmoside 紫草氰苷	$C_{14}H_{19}NO_8$	329.303	NA	C17771	NA	NA	NA	NA	NULL
136656-07-0	1228026	911571	682217	Timosaponin B II 知母皂苷B II	$C_{45}H_{76}O_{19}$	921.073	NA	NA	NA	NA	NA	NA	NA
638-23-3	1217226	1310326	1143167	Carbocysteine	$C_5H_9NO_4S$	179.194	NA	C03727	NA	NA	NA	NA	NULL
31721-94-5	1211689	1043920	1107524	5,7-Dihydroxychromone 5,7-二羟基色原酮	$C_9H_6O_4$	178.141	HMDB0032950	C09001	Organoheterocyclic compounds	Benzopyrans	1-benzopyrans	1-benzopyrans	NA
487-26-3	1203843	1606333	1837972	Flavanone 黄烷酮	$C_{15}H_{12}O_2$	224.255	NA	NA	NA	NA	NA	NA	NA
476-28-8	1186591	836841	1227519	Lycorine 石蒜碱	$C_{16}H_{17}NO_4$	287.31	NA	C08532	NA	NA	NA	Alkaloids	map00950 Isoquinoline alkaloid biosynthesis; map01063 Biosynthesis of alkaloids derived from shikimate pathway
2188-68-3	1186591	836841	1227519	Lycorine chloride 盐酸石蒜碱	$C_{16}H_{18}ClNO_4$	323.77	NA	NA	NA	NA	NA	NA	NA
123-11-5	1167204	1941765	1162793	Anisic aldehyde 对甲氧基苯甲醛	$C_8H_8O_2$	136.148	HMDB0029686	C10761	Benzenoids	Benzene and substituted derivatives	Benzoyl derivatives	Benzene and derivatives	NA
118-41-2	1161770	863793	924954	3,4,5-Trimethoxybenzoic acid	$C_{10}H_{12}O_5$	212.199	HMDB0033839	NA	Benzenoids	Benzene and substituted derivatives	Benzoic acids and derivatives	Benzene and derivatives	NA
357-70-0	1148299	1116937	986464	Galanthamine 加兰他敏	$C_{17}H_{21}NO_3$	287.353	HMDB0014812	C08526	Alkaloids and derivatives	Amaryllidaceae alkaloids	Galanthamine-type amaryllidaceae alkaloids	Alkaloids and derivatives	NA
55418-52-5	1147871	1150025	1114304	Piperonyl acetone	$C_{11}H_{12}O_3$	192.211	HMDB0041481	NA	Organoheterocyclic compounds	Benzodioxoles	null	Benzodioxoles	NA
19453	1129309	986771	911148	Galanthamine hydrobromide	$C_{17}H_{22}BrNO_3$	368.266	NA	NA	NA	NA	NA	NA	NA

470-17-7	1128605	956247	1066782	Isoalantolactone 异土木香内酯	C15H20O2	232.318	HMDB0035934	C09484	Lipids and lipid-like molecules	Prenol lipids	Terpene lactones	Terpenoids	NA
501-98-4	1126134	754240	213782	p-Coumaric acid 对羟基肉桂酸	C9H8O3	164.158	HMDB0002035	NA	Phenylpropanoids and polyketides	Cinnamic acids and derivatives	Hydroxycinnamic acids and derivatives	Polyketides[PK]	NA
96-82-2	1125780	1284587	795633	Lactobionic acid	C12H22O12	358.296	NA	NA	NA	NA	NA	NA	NA
11013-97-1	1116289	1218732	1056221	Methyl hesperidin 甲基橙皮苷	C29H36O15	624.587	NA	C17393	NA	NA	NA	NA	NULL
487-52-5	1101397	1145079	1127833	BUTEIN	C15H12O5	272.25	NA	C08578	NA	NA	NA	Flavonoids	map00941 Flavonoid biosynthesis
7400-08-0	1099700	560332	14712	p-Hydroxycinnamic acid 对羟基肉桂酸	C9H8O3	164.158	NA	NA	NA	NA	NA	NA	NA
133164-11-1	1095082	1140679	902638	Oxypeucedan hydrate 水合氧化前胡素	C16H16O6	304.295	NA	NA	NA	NA	NA	NA	NA
1241-87-8	1092918	1492638	1212004	1-Caffeoylquinic acid 1-咖啡酰奎宁酸	C16H18O9	354.309	HMDB0030652	NA	Organic oxygen compounds	Organooxygen compounds	Alcohols and polyols	Alcohols	NA
519-23-3	1085237	1003716	889409	Ellipticine 椭圆玫瑰树碱	C17H14N2	246.307	NA	C09154	NA	NA	NA	Alkaloids	NULL
1190-49-4	1076111	922747	992379	L-Homocitrulline	C7H15N3O3	189.212	HMDB0000679	C02427	Organic acids and derivatives	Carboxylic acids and derivatives	Amino acids, peptides, and analogues	Amino acids, peptides, and analogues	NA
61825-98-7	1074550	942209	1119511	Imperialine 西贝母碱	C27H43NO3	429.635	NA	C10808	NA	NA	NA	Alkaloids	NULL
68-95-1	1073425	1107680	1087517	N-Acetyl-L-Proline	C7H11NO3	157.167	HMDB0094701	NA	Organic acids and derivatives	Carboxylic acids and derivatives	Amino acids, peptides, and analogues	Amino acids, peptides, and analogues	NA
18296	1073210	1028093	947996	Dexamethasone	C22H29FO5	392.461	HMDB0015364	C15643	Lipids and lipid-like molecules	Steroids and steroid derivatives	Hydroxysteroids	Steroids and derivatives	NA
5574-24-3	1069644	883770	1016684	Oxoglaucine 氧海罂粟碱	C20H17NO5	351.353	HMDB0029337	NA	Organic acids and derivatives	Carboxylic acids and derivatives	Amino acids, peptides, and analogues	Alkaloids and derivatives	NA
134-96-3	1063967	999811	986019	Syringaldehyde	C9H10O4	182.173	NA	NA	NA	NA	NA	NA	NA
102518-79-6	1055205	980524	944041	(-)-Huperzine A (-)-石杉碱甲	C15H18N2O	242.32	NA	NA	NA	NA	NA	NA	NA
6384-92-5	1051949	1079802	1030358	N-Methyl-D-aspartic acid	C5H9NO4	147.129	HMDB0002393	NA	Organic acids and derivatives	Carboxylic acids and derivatives	Amino acids, peptides, and analogues	Amino acids, peptides, and analogues	NA
486-56-6	1051315	1056783	1091460	(-)-Cotinine (-)-可替宁	C10H12N2O	176.215	HMDB0001046	NA	Organoheterocyclic compounds	Pyridines and derivatives	Pyrrolidinylpyridines	Pyridine and derivatives	NA
116183-66-5	1047135	1277052	920885	Complanatuside 沙苑子苷	C28H32O16	624.544	NA	NA	NA	NA	NA	NA	NA
57420-46-9	1043347	958860	856566	8-O-Acetyl shanzhiside methyl ester 8-O-乙酰山栀苷甲酯	C19H28O12	448.418	NA	NA	NA	NA	NA	NA	NA
2980-32-7	1041463	749546	754229	Tetrahydroxyxanthone 去甲基雏叶龙胆酮	C13H8O6	260.199	NA	C10056	NA	NA	NA	Polyketides[PK]	NULL
495-85-2	1036701	825203	1100551	Methysticin 麻醉椒苦素	C15H14O5	274.269	HMDB0033590	C09952	Phenylpropanoids and polyketides	Kavalactones	null	Polyketides[PK]	NA
26904-64-3	1035916	933468	1249672	Oxysophocarpine 氧化槐果碱	C15H22N2O2	262.347	NA	NA	NA	NA	NA	NA	NA
5471-51-2	1025697	1067684	1026503	Raspberry ketone	C10H12O2	164.201	HMDB0033723	NA	Benzenoids	Phenols	1-hydroxy-2-unsubstituted benzenoids	Phenols and derivatives	NA
73069-25-7	1024495	1127182	1095341	Praeruptorin A 白花前胡甲素	C21H22O7	386.395	NA	NA	NA	NA	NA	NA	NA
5302-45-4	1020659	888155	853858	3-[(Carboxycarbonyl)amino]-L-alanine 三七素	C5H8N2O5	176.127	HMDB0029402	C04209	Organic acids and derivatives	Carboxylic acids and derivatives	Amino acids, peptides, and analogues	Amino acids, peptides, and analogues	NA
4674-50-4	995645	910661	993938	(+)-Nootkatone 诺卡酮	C15H22O	218.335	HMDB0013687	C17914	Lipids and lipid-like molecules	Prenol lipids	Sesquiterpenoids	Terpenoids	NA

6893-26-1	988204	997786	1067925	D-Glutamic acid	C5H9NO4	147.129	HMDB0003339	C00217	Organic acids and derivatives	Carboxylic acids and derivatives	Amino acids, peptides, and analogues	Amino acids, peptides, and analogues	NA
65497-07-6	987160	1614677	1381096	Esculentoside A 商陆皂苷甲	C42H66O16	826.964	HMDB0034637	NA	Lipids and lipid-like molecules	Prenol lipids	Triterpenoids	Terpenoids	NA
99-50-3	984685	839519	1049922	Protocatechuic acid 原儿茶酸	C7H6O4	154.12	HMDB0001856	C00230	Benzenoids	Benzene and substituted derivatives	Benzoic acids and derivatives	Benzene and derivatives	NA
118-00-3	976444	1025757	806711	Guanosine 鸟苷	C10H13N5O5	283.241	HMDB0000133	C00387	Nucleosides, nucleotides, and analogues	Purine nucleosides	null	Purines and derivatives	NA
5041-82-7	974195	658206	776956	Isorhamnetin 3-O-glucoside	C22H22O12	478.403	NA	NA	NA	NA	NA	NA	NA
469-83-0	972483	905654	913972	Cafestol 咖啡醇	C20H28O3	316.435	HMDB0035710	C09066	Organoheterocyclic compounds	Naphthofurans	null	Naphthofurans	NA
13657-68-6	970799	903580	1303001	Curdione 莪术二酮	C15H24O2	236.35	HMDB0034714	C17493	Lipids and lipid-like molecules	Prenol lipids	Sesquiterpenoids	Terpenoids	NA
480-64-8	963355	967646	865605	Orsellinic acid 苔色酸	C8H8O4	168.147	NA	C01839	NA	NA	NA	Polyketides[PK]	NULL
22059-21-8	956979	809486	774766	1-Aminocyclopropanecarboxylic acid	C4H7NO2	101.104	HMDB0036458	C01234	Organic acids and derivatives	Carboxylic acids and derivatives	Amino acids, peptides, and analogues	Amino acids, peptides, and analogues	NA
529-53-3	954739	1291233	1462808	Scutellarein 高黄芩素	C15H10O6	286.236	NA	C10184	Phenylpropanoids and polyketides	Flavonoids	Flavones	Flavonoids	NULL
378-44-9	940675	982683	830278	Betamethasone	C22H29FO5	392.461	HMDB0014586	C06848	Lipids and lipid-like molecules	Steroids and steroid derivatives	Hydroxysteroids	Steroids and derivatives	NA
527-95-7	939433	829746	824916	Herbacetin 草质素	C15H10O7	302.236	NA	NA	NA	NA	NA	NA	NA
520-18-3	938351	1106770	877175	Kaempferol 山奈酚	C15H10O6	286.236	HMDB0005801	C05903	Phenylpropanoids and polyketides	Flavonoids	Flavones	Flavonoids	NA
21709-90-0	937095	1005273	974370	N-Propionylglycine	C5H9NO3	131.13	HMDB0000783	NA	Organic acids and derivatives	Carboxylic acids and derivatives	Amino acids, peptides, and analogues	Amino acids, peptides, and analogues	NA
121-33-5	934921	931527	1160639	Vanillin 香兰素	C8H8O3	152.147	HMDB0012308	C00755	Benzenoids	Phenols	Methoxyphenols	Phenols and derivatives	NA
121-34-6	926795	785354	833776	Vanillic acid 香草酸	C8H8O4	168.147	HMDB0000484	C06672	Benzenoids	Benzene and substituted derivatives	Benzoic acids and derivatives	Benzene and derivatives	NA
33457-62-4	926501	828066	872183	Alnustone 桤木酮	C19H18O	262.346	HMDB0031664	NA	Phenylpropanoids and polyketides	Diarylheptanoids	Linear diarylheptanoids	Polyketides[PK]	NA
2948-76-7	912911	1041760	1009871	Fisetinidin chloride	C15H11ClO5	306.698	NA	NA	NA	NA	NA	NA	NA
23800-56-8	903562	918896	1436860	Pogostone 广藿香酮	C12H16O4	224.253	HMDB0030703	NA	Organic oxygen compounds	Organooxygen compounds	Carbonyl compounds	Carbonyl compounds	NA
57817-89-7	902337	681680	909347	Stevioside 甜菊糖	C38H60O18	804.872	HMDB0034945	C09189	Lipids and lipid-like molecules	Prenol lipids	Terpene glycosides	Terpenoids	NA
500-64-1	892054	1013993	1386451	L-Kawain 醉椒素	C14H14O3	230.259	HMDB0034212	C09947	Phenylpropanoids and polyketides	Kavalactones	null	Polyketides[PK]	NA
631-01-6	871938	808422	767216	Quillaic acid 皂皮酸	C30H46O5	486.683	NA	C08972	NA	NA	NA	Terpenoids	NULL
124-20-9	871652	954317	879407	Spermidine	C7H19N3	145.246	HMDB0001257	C00315	Organic nitrogen compounds	Organonitrogen compounds	Amines	Amines	NA
611-40-5	864610	1128873	771670	Tectoridin 射干苷	C22H22O11	462.404	NA	C10533	NA	NA	NA	Flavonoids	NULL
67604-48-2	858294	958315	681029	Naringenin	C15H12O5	272.253	NA	NA	NA	NA	NA	NA	NA
482-35-9	858026	601348	194015	Isoquercitrin 异槲皮苷	C21H20O12	464.376	HMDB0037362	NA	Phenylpropanoids and polyketides	Flavonoids	Flavonoid glycosides	Flavonoids	NA
3211-76-5	852352	995521	1026465	L-(+)-Selenomethionine	C5H11NO2Se	196.106	HMDB0003966	C05335	Organic acids and derivatives	Carboxylic acids and derivatives	Amino acids, peptides, and analogues	Amino acids, peptides, and analogues	NA
555-66-8	852015	772878	690718	6-Shogaol 6-姜烯酚	C17H24O3	276.371	NA	C10494	Benzenoids	Phenols	Methoxyphenols	Polyketides[PK]	NULL
116-31-4	850874	874273	859930	All trans-Retinal	C20H28O	284.436	HMDB0001358	C00376	Lipids and lipid-like molecules	Prenol lipids	Retinoids	Prenol lipids[PR]	NA
80681-45-4	846344	560414	853621	Prim-O-glucosylcimifugin	C22H28O11	468.451	NA	NA	NA	NA	NA	NA	NA

				升麻素苷									
498-40-8	844120	740466	665089	L-Cysteic acid	C3H7NO5S	169.156	HMDB0002757	NA	Organic acids and derivatives	Carboxylic acids and derivatives	Amino acids, peptides, and analogues	Amino acids, peptides, and analogues	NA
29838-67-3	839579	872285	790673	Astilbin 落新妇苷	C21H22O11	450.393	HMDB0033850	C17449	Phenylpropanoids and polyketides	Flavonoids	Flavonoid glycosides	Flavonoids	NA
125675-09-4	828420	556581	602450	Blinin 苦蒿素	C22H32O6	392.486	NA	NA	NA	NA	NA	NA	NA
26146-27-0	819599	781065	845270	Lindenenol 乌药醇	C15H18O2	230.302	NA	C16988	NA	NA	NA	NA	NULL
13752-84-6	804851	774283	667725	D-Erythronic acid	C4H8O5	136.1033	HMDB0000613	NA	Organic oxygen compounds	Organooxygen compounds	Carbohydrates and carbohydrate conjugates	Carbohydrates	NA
10597-60-1	797630	668380	718749	3,4-Dihydroxy-phenylethanol 羟基酪醇	C8H10O3	154.163	HMDB0005784	NA	Benzenoids	Phenols	Tyrosols and derivatives	Phenols and derivatives	NA
20033	796944	804430	759346	L-(-)-Nicotine	C10H14N2	162.232	HMDB0001934	C00745	Organoheterocyclic compounds	Pyridines and derivatives	Pyrrolidinylpyridines	Pyridine and derivatives	NA
38183-03-8	792853	733929	898151	7,8-Dihydroxyflavone	C15H10O4	254.24	NA	NA	NA	NA	NA	NA	NA
30964-13-7	787029	752107	919242	Cynarin	C25H24O12	516.451	HMDB0030093	C10445	Organic oxygen compounds	Organooxygen compounds	Alcohols and polyols	Alcohols	NA
5505-63-5	786943	530052	783018	D-Mannosamine	C6H14ClNO5	215.632	NA	NA	NA	NA	NA	NA	NA
81-27-6	782267	748306	742468	Sennoside A 番泻苷 A	C42H38O20	862.739	HMDB0034317	C10404	Benzenoids	Anthracenes	Anthracenecarboxylic acids and derivatives	Benzene and derivatives	NA
27876-94-4	763689	741677	690724	Crocetin 藏红花酸	C20H24O4	328.402	HMDB0035098	C08588	Lipids and lipid-like molecules	Prenol lipids	Diterpenoids	Terpenoids	NA
128-57-4	762364	644015	736357	Sennoside B 番泻苷B	C42H38O20	862.739	HMDB0002783	C13526	Benzenoids	Anthracenes	Anthracenecarboxylic acids and derivatives	Benzene and derivatives	NA
700-58-3	761947	741753	847839	2-Adamantanone 2-金刚烷酮	C10H14O	150.218	NA	NA	NA	NA	NA	NA	NA
313-67-7	748476	894910	716162	Aristolochic acid 马兜铃酸	C17H11NO7	341.27	NA	C08469	NA	NA	NA	Alkaloids	NULL
3416-24-8	747065	740259	787396	D-(+)-Glucosamine	C6H13NO5	179.171	HMDB0001514	C00329	Organic oxygen compounds	Organooxygen compounds	Carbohydrates and carbohydrate conjugates	Carbohydrates	NA
70831-56-0	743236	769675	668697	Cichoric acid 菊苣酸	C22H18O12	474.371	HMDB0002375	C10437	Organic acids and derivatives	Carboxylic acids and derivatives	Tetracarboxylic acids and derivatives	Organic acids	NA
63-91-2	741782	995988	688412	L-Phenylalanine L-苯丙氨酸	C9H11NO2	165.189	HMDB0000159	C00079	Organic acids and derivatives	Carboxylic acids and derivatives	Amino acids, peptides, and analogues	Amino acids	NA
87-78-5	739281	807303	916214	Mannitol 甘露醇	C6H14O6	182.172	NA	NA	NA	NA	NA	NA	NA
552-58-9	735839	774216	404262	Eriodictyol 圣草酚	C15H12O6	288.252	HMDB0005810	C05631	Phenylpropanoids and polyketides	Flavonoids	Flavans	Flavonoids	NA
34520	730071	693257	999157	D-(-)-Synephrine	C9H13NO2	167.205	HMDB0004826	C04548	Benzenoids	Phenols	1-hydroxy-2-unsubstituted benzenoids	Phenols and derivatives	NA
110-15-6	725758	670288	655132	Succinic acid	C4H6O4	118.088	HMDB0000254	C00042	Organic acids and derivatives	Carboxylic acids and derivatives	Dicarboxylic acids and derivatives	Organic acids	NA
149-91-7	725485	699567	642095	Gallic acid 没食子酸	C7H6O5	170.12	HMDB0005807	C01424	Benzenoids	Benzene and substituted derivatives	Benzoic acids and derivatives	Benzene and derivatives	NA
479-13-0	721515	548185	589622	Coumestrol	C15H8O5	268.221	HMDB0002326	C10205	Phenylpropanoids and polyketides	Isoflavonoids	Coumestans	Coumarins and derivatives	NA
501-36-0	714180	751737	765087	Resveratrol 白藜芦醇	C14H12O3	228.243	HMDB0003747	C03582	Phenylpropanoids and polyketides	Stilbenes	null	Polyketides[PK]	NA
524-12-9	713607	784213	589774	Wedelolactone 蟛蜞菊内酯	C16H10O7	314.246	NA	C10541	NA	NA	NA	Flavonoids	NULL
480-20-6	704923	838709	702282	DIHYDROKAEMPFEROL	C15H12O6	288.252	HMDB0030847	C00974	Phenylpropanoids and polyketides	Flavonoids	Flavans	Flavonoids	NA
35825-57-1	700099	803169	703464	Cryptotanshinone 隐丹参酮	C19H20O3	296.36	HMDB0035220	NA	Lipids and lipid-like molecules	Prenol lipids	Diterpenoids	Terpenoids	NA

28860-95-9	698613	620477	569451	S-(-)-Carbidopa 卡比多巴	C10H14N2O4	226.23	HMDB0014336	NA	Phenylpropanoids and polyketides	Phenylpropanoic acids	null	Phenylpropanoic acids	NA
25274-27-5	697992	597408	675977	Aristolone 马兜铃酮	C15H22O	218.335	NA	NA	NA	NA	NA	NA	NA
584-85-0	693725	736974	647222	L-Anserine	C10H16N4O3	240.259	HMDB0000194	C01262	Organic acids and derivatives	Peptidomimetics	Hybrid peptides	Organic acids	NA
32449-92-6	691092	534628	595470	D-Glucuro-3,6-lactone	C6H8O6	176.124	HMDB0006355	C02670	Organoheterocyclic compounds	Furofurans	Isosorbides	Isosorbides	NA
54261-98-2	684342	581228	434611	Stachyose 水苏糖	C24H42O21	720.623	NA	NA	NA	NA	NA	NA	NA
130-01-8	678671	482772	715381	Senecionine 千里光碱	C18H25NO5	335.395	NA	C06176	NA	NA	NA	Alkaloids	map00960 Tropane, piperidine and pyridine alkaloid biosynthesis; map01064 Biosynthesis of alkaloids derived from ornithine, lysine and nicotinic acid; map01110 Biosynthesis of secondary metabolites
464-49-3	678277	440041	446612	(+)-Camphor	C10H16O	152.233	HMDB0059838	C00808	Lipids and lipid-like molecules	Prenol lipids	Monoterpenoids	Terpenoids	NA
23541-50-6	677977	585143	529962	Daunorubicin hydrochloride 盐酸柔红霉素	C27H30ClNO10	563.981	NA	NA	NA	NA	NA	NA	NA
93-15-2	675907	587578	616397	Methyl eugenol 甲基丁香酚	C11H14O2	178.228	HMDB0031864	C10454	Benzenoids	Benzene and substituted derivatives	Methoxybenzenes	Benzene and derivatives	NA
14641-93-1	675039	731351	772232	Lactose 乳糖	C12H22O11	342.296	NA	NA	NA	NA	NA	NA	NA
608-07-1	673583	745846	1170737	5-Methoxytryptamine	C11H14N2O	205.21	HMDB0004095	C05659	Organoheterocyclic compounds	Indoles and derivatives	Tryptamines and derivatives	Amines and derivatives	NA
518-69-4	669694	633007	673920	Corydaline 延胡索甲素	C22H27NO4	369.454	NA	C15530	NA	NA	NA	Alkaloids	map00950 Isoquinoline alkaloid biosynthesis; map01110 Biosynthesis of secondary metabolites
22007-72-3	663387	583184	578285	Quercetin 7-rhamnoside 田基黄苷	C21H20O11	448.377	NA	NA	NA	NA	NA	NA	NA
28978-03-2	663061	626581	673307	Persicoside	C23H26O11	478.4459	HMDB0037482	NA	Phenylpropanoids and polyketides	Flavonoids	Flavonoid glycosides	Flavonoids	NA
24939-16-0	662166	502070	624601	Bisdemethoxy-curcumin 双脱氧基姜黄素	C19H16O4	308.328	HMDB0002114	C17743	Phenylpropanoids and polyketides	Diarylheptanoids	Linear diarylheptanoids	Polyketides[PK]	NA
72361-67-2	654067	593393	612190	Laetanine 四氢二甲氧基二苯并喹啉二醇	C18H19NO4	313.348	NA	NA	NA	NA	NA	NA	NA
112-43-6	646478	613316	617793	10-Undecen-1-ol	C11H22O	170.292	HMDB0031016	NA	Lipids and lipid-like molecules	Fatty Acyls	Fatty alcohols	Fatty acyls[FA]	NA
3943-74-6	645343	606996	551847	Methyl vanillate 香草酸甲酯	C9H10O4	182.173	HMDB0240266	NA	Benzenoids	Benzene and substituted derivatives	Benzoic acids and derivatives	Benzene and derivatives	NA
18490-95-4	645110	652532	771746	Brevifolincarboxylic acid 短叶苏木酚酸	C13H8O8	292.198	NA	NA	NA	NA	NA	NA	NA
104-01-8	644519	631087	660882	4-Methoxy-phenylacetic acid 4-甲氧基苯乙酸	C9H10O3	166.174	HMDB0002072	NA	Benzenoids	Phenol ethers	Anisoles	Benzene and derivatives	NA
634-97-9	644425	600216	637749	Pyrrole-2-carboxylic acid	C5H5NO2	111.099	HMDB0004230	NA	Organoheterocyclic compounds	Pyrroles	Pyrrole carboxylic acids and derivatives	Pyrrole carboxylic acids and derivatives	NA
138-52-3	643580	637698	612078	D(-)-Salicin 水杨苷	C13H18O7	286.278	HMDB0003546	C01451	Organic oxygen compounds	Organooxygen compounds	Carbohydrates and carbohydrate conjugates	Carbohydrates	NA
15687-27-1	641428	429104	575244	Ibuprofen	C13H18O2	206.281	HMDB0001925	C01588	Phenylpropanoids and	Phenylpropanoic acids	null	Phenylpropanoic acids	NA

									polyketides				
861691-37-4	640163	583339	532192	2''-O-β-L-Galactopyranosylorientin 荭草素-2''-O-β-L半乳糖苷	C27H30O16	610.518	NA	NA	NA	NA	NA	NA	NA
20516-23-8	639416	657868	561420	Peucedanol 白花前胡醇	C14H16O5	264.274	NA	NA	NA	NA	NA	NA	NA
88509-91-5	639387	556364	689402	Dichotomitin 白射干素	C18H14O8	358.299	NA	NA	NA	NA	NA	NA	NA
93-35-6	626567	619278	790861	7-Hydroxycoumarin 7-羟基香豆素	C9H6O3	162.142	HMDB0029865	C09315	Phenylpropanoids and polyketides	Coumarins and derivatives	Hydroxycoumarins	Coumarins and derivatives	NA
19773-24-1	622616	666992	660213	Peimisine 贝母辛	C27H41NO3	427.619	NA	NA	NA	NA	NA	NA	NA
528-48-3	619708	629737	565951	Fisetin 漆黄素	C15H10O6	286.236	NA	C10041	NA	NA	NA	Flavonoids	NULL
531-28-2	618918	595845	640230	Androsin 草夹竹桃苷	C15H20O8	328.315	NA	NA	NA	NA	NA	NA	NA
476-69-7	612819	598461	577984	Corydine 紫堇定酚	C20H23NO4	341.401	NA	NA	NA	NA	NA	NA	NA
55399-93-4	609247	606330	567139	4-Hydroxyisoleucine 羟基异亮氨酸	C6H13NO3	147.172	NA	C20802	NA	NA	NA	NA	NULL
120-47-8	604389	406368	285013	Ethylparaben 尼泊金乙	C9H10O3	166.174	HMDB0032573	NA	Benzenoids	Benzene and substituted derivatives	Benzoic acids and derivatives	Benzene and derivatives	NA
751-03-1	603441	471669	494730	Obacunone 黄柏酮	C26H30O7	454.512	HMDB0035858	C08775	Lipids and lipid-like molecules	Prenol lipids	Triterpenoids	Terpenoids	NA
64-77-7	600018	558134	505594	Tolbutamide	C12H18N2O3S	270.348	HMDB0015256	C07148	Benzenoids	Benzene and substituted derivatives	Benzenesulfonamides	Benzene and derivatives	NA
5699-54-7	594908	597195	720259	DL-β-Leucine	C6H13NO2	131.173	HMDB0003640	NA	Organic acids and derivatives	Carboxylic acids and derivatives	Amino acids, peptides, and analogues	Amino acids, peptides, and analogues	NA
